# Supplementary material for: Gαq activation modulates autophagy by promoting mTORC1 signaling
Source: Nat Commun. 2021 Jul 27;12:4540. doi: 10.1038/s41467-021-24811-4 (PMC8316552; doi:10.1038/s41467-021-24811-4)
Supplement: Supplementary file 1 — Supplementary Information [file 41467_2021_24811_MOESM1_ESM.pdf]

Supplementary Figures, Legends and Table 1

Gαq activation modulates autophagy by promoting mTORC1 signaling

Sofía Cabezudo[1,2,3,4,&], Maria Sanz-Flores[1,2,&] , Alvaro Caballero[1,2], Inmaculada Tasset[5], Elena Rebollo[7], Antonio Diaz[5], Anna M. Aragay[6], Ana María Cuervo[5], Federico Mayor jr[1,2,3,*] and Catalina Ribas[1,2,3,*]

[1]Departamento de Biología Molecular and Centro de Biología Molecular "Severo Ochoa" (UAM-CSIC), 28049 Madrid, Spain;
[2]Instituto de Investigación Sanitaria La Princesa, 28006 Madrid, Spain;
[3]CIBER de Enfermedades Cardiovasculares, ISCIII (CIBERCV), 28029 Madrid, Spain,
[4] Structural Biology Program, Spanish National Cancer Research Centre (CNIO), 28029 Madrid, Spain. [5] Department of Developmental and Molecular Biology and; Institute for Aging Research, Albert Einstein College of Medicine, Bronx, NY, USA.
[6] Department of Biology, Molecular Biology Institute of Barcelona (IBMB), Spanish National Research Council (CSIC), Barcelona 08028, Spain. [7] Molecular Imaging Platform (MIP), Molecular Biology Institute of Barcelona (IBMB), Spanish National Research Council (CSIC), Barcelona 08028, Spain

[&] Equal contribution
* Corresponding authors. Contact information:
Centro de Biología Molecular "Severo Ochoa",
C/ Nicolás Cabrera 1, Universidad Autónoma de Madrid, 28049 Madrid, Spain.
cribas@cbm.csic.es; fmayor@cbm.csic.es
Phone: 34-91-1964728/4626; Fax: 34-91-196-4420

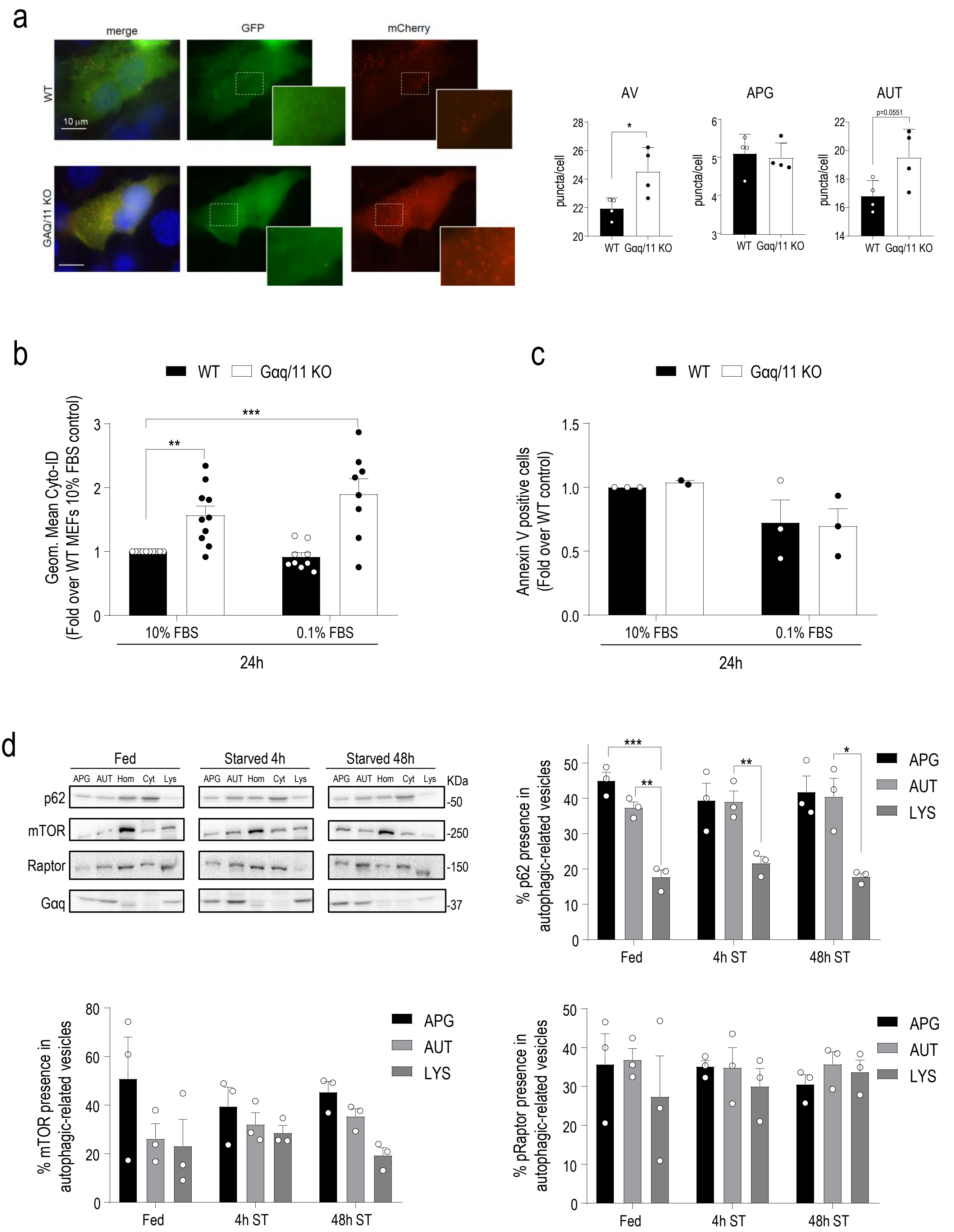

Figure Supp 1

Supplementary Fig.1. Cells lacking Gαq/11 display a higher autophagic flux while maintaining unaltered its viability. a) Representative images of WT and Gαq/11 KO MEFs transduced with a lentivirus expressing the tandem reporter mCherry-GFP-LC3. Nuclei are stained in blue with DAPI. Single and merged channels are shown. Insets: higher magnification. Quantification of the number of autophagic vacuoles (AV), autophagosomes (APG) or Autolysosomes (AUT) per cell. All values are mean ± SEM of triplicate wells and quantification were done in at least 2.500 cells per condition using high content microscopy in 4 independent experiments. AV: WT vs. Gαq/11KO, *p*-value=0.0314. b) Autophagic vacuoles of WT and Gαq/11 KO MEFs starved for 24h in 0.1% FBS were quantified using the Cyto-ID Autophagy detection Kit, according to manufacturer's instructions. Data (Geom. Mean Fluorescence ± SEM of 10 (WT and Gαq/11KO in 10%FBS), 9 (WT in 0.1%FBS) and 8 (Gαq/11KO in 0.1%FBS) independent experiments) were represented as the fold-induction over WT MEFs at basal conditions. WT vs. Gαq/11KO in 10%FBS, *p*-value=0.0010; WT(10%FBS) vs. Gαq/11KO(0.1%FBS), *p*-value=0.0006. c) Apoptotic cell death was quantified using PE Annexin V Apoptosis Detection Kit I, according to manufacturer's instructions. WT and Gαq/11 KO MEFs were starved for 24h with 0.1% FBS. Samples were analyzed by flow cytometry. Early apoptosis data were represented as fold-induction with respect to the WT control (mean ± SEM of 3 independent experiments). d) Immunoblot analysis of p62, mTOR, Raptor and Gαq in autophagy-related organelles isolated from livers of fed or 4 and 48h starved rats. Homogenate (HOM); cytosol (Cyt); autophagosomes (APG); autolysosomes (AUT); lysosomes (Lys). A representative blot is shown. Densitometric quantification values are expressed as the percentage of total protein associated to autophagic/lysosomal compartment in each of these compartments. Values are the mean ± SEM of 3 independent experiments. APG(Fed) vs. LYS(Fed), *p*-value=0.0010; AUT(Fed) vs. LYS(Fed), *p*-value=0.0017; AUT(4h ST) vs. LYS(4h ST), *p*-value=0.087; AUT(48h ST) vs. LYS(48h ST), *p*-value=0.0126. Statistical significance was analyzed using unpaired *t*-test. For all *p*-values, *p<0.05, **p<0.01, ***p<0.001. Source data are provided as a Source Data file.

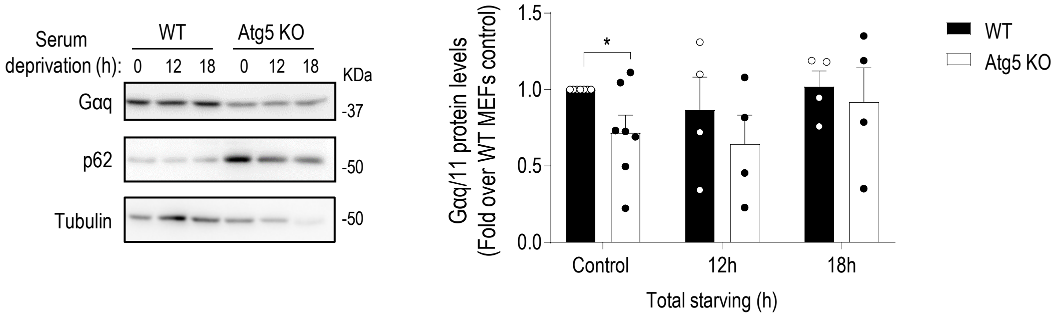

Supplementary Figure 2

Supplementary Fig.2. Gαq protein is not a substrate of macroautophagy. Immunoblot for Gαq protein levels in WT and Atg5 KO MEFs starved for 12-18h with serum-free medium. p62 was used as a known autophagy substrate control. Tubulin was used as loading control. Data (mean ± SEM of 4(12h and 18h) or 7(control) independent experiments) were normalized and expressed as fold-induction with respect to the control condition. Statistical significance was analyzed using two-sided unpaired *t*-test. WT vs. Atg5 KO, control condition, *p*-value=0.0308. A representative blot is shown. Source data are provided as a Source Data file.

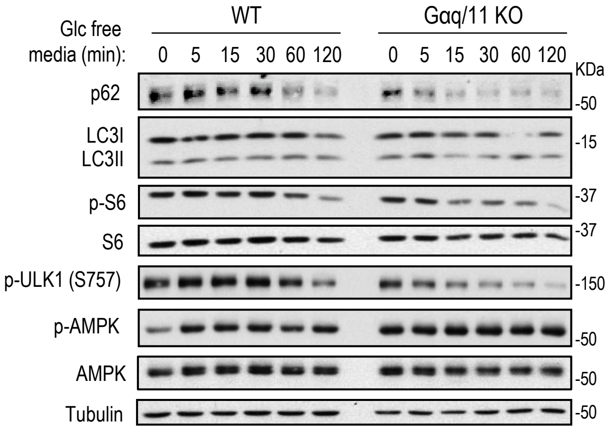

Supplementary Figure 3

Supplementary Fig.3. Cells lacking Gαq/11 display an earlier and prolonged autophagic response under glucose-free conditions along with an altered mTORC1 activation status. WT and Gαq/11 KO MEFs were maintained in presence or absence of glucose during the indicated times. The modulation of autophagy, mTORC1 and AMPK pathways was analyzed as in Fig. 2. A representative blot of 4 independent experiments is shown.

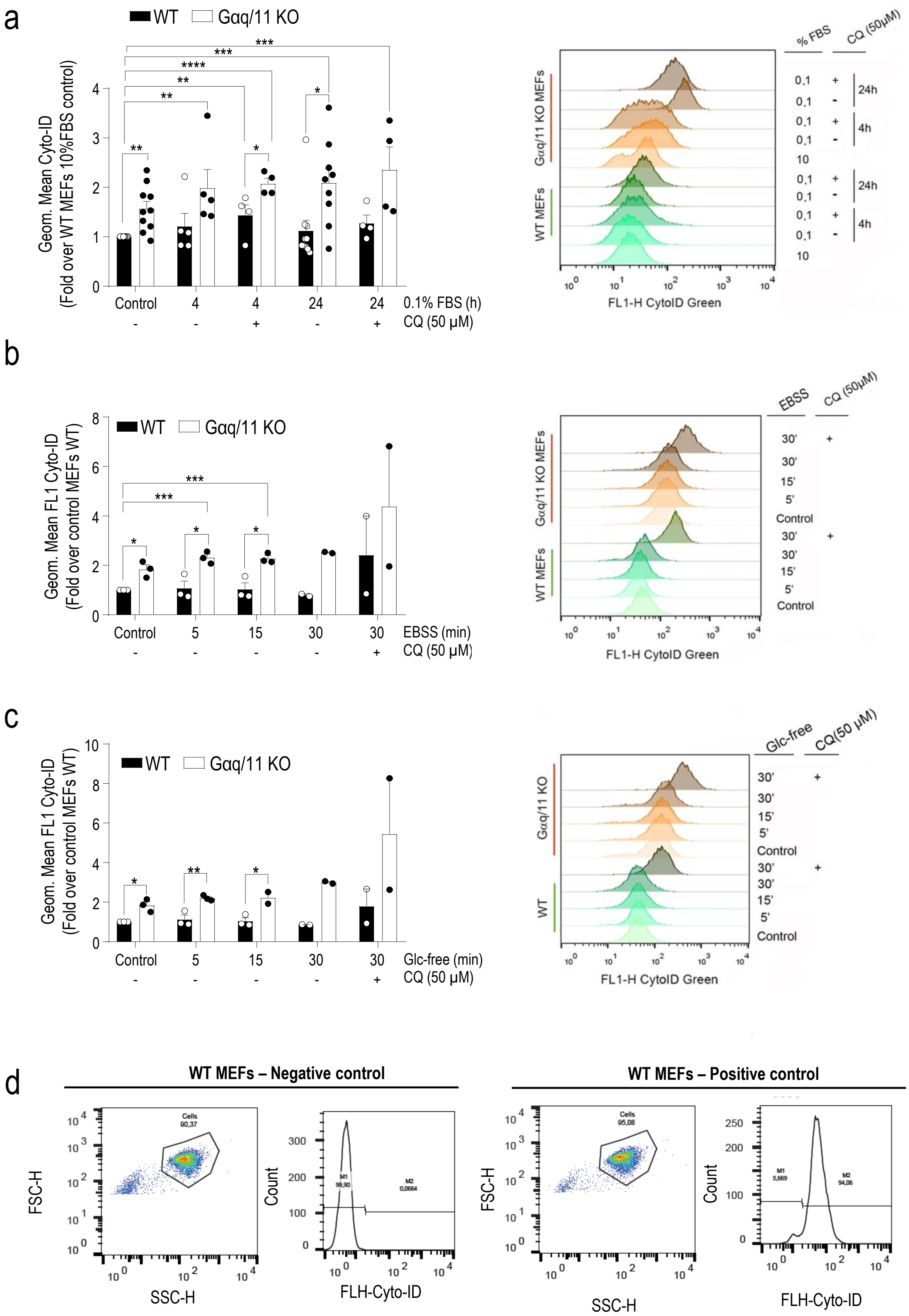

Supplementary Figure 4

Supplementary Fig.4. Cells lacking Gαq/11 display an earlier and prolonged autophagic response under different nutrient stress conditions. a) WT and Gαq/11 KO MEFs were maintained in presence or absence of serum during the indicated times. Data (Geom. mean fluorescence ± SEM of n=10 (control), n=5 (t=4), n=4 (t=4 and t=24, +CQ) and n=10 (WT, t=24 -CQ) and n=9 (Gαq/11 KO, t=24 -CQ) were represented as the fold-induction over WT MEFs at basal conditions. WT vs. Gαq/11 KO MEFs, control, *p*-value=0.0010; WT vs. Gαq/11 KO MEFs, t=4, +CQ, *p*-value=0.0371; WT vs. Gαq/11 KO MEFs, t=24, -CQ, *p*-value=0.0134; WT(control) vs. Gαq/11 KO(t=4, -CQ) MEFs, *p*-value=0.0019; WT(control) vs. WT(t=4, +CQ) MEFs, *p*-value=0.0044; WT(control) vs. Gαq/11 KO(t=4, +CQ) MEFs, *p*-value<0.0001; WT(control) vs. Gαq/11 KO(t=24, -CQ) MEFs, *p*-value=0.0008; WT(control) vs. Gαq/11 KO(t=24, +CQ) MEFs, *p*-value=0.0003. b) WT and Gαq/11 KO MEFs were maintained in presence or absence of amino acids during the indicated times. Data (Geom. mean fluorescence ± SEM of n=3 (control, t=5 and t=15 conditions) and n=2 (t=30 ±CQ conditions) experiments) were represented as the fold-induction over WT MEFs at basal conditions. WT vs. Gαq/11 KO MEFs, control, *p*-value=0.0110; WT vs. Gαq/11 KO MEFs, t=5, *p*-value=0.0186; WT vs. Gαq/11 KO MEFs, t=15, *p*-value=0.0122; WT(control) vs. Gαq/11 KO(t=5) MEFs, *p*-value=0.0007; WT(control) vs. Gαq/11 KO(t=15) MEFs, *p*-value=0.0004. c) WT and Gαq/11 KO MEFs were maintained in presence or absence of glucose during the indicated times. Data (Geom. mean fluorescence ± SEM of n=3 (control, t=5 and t=15 conditions) and n=2 (t=30 ±CQ conditions) experiments) were represented as the fold-induction over WT MEFs at basal conditions. WT vs. Gαq/11 KO MEFs, control, *p*-value=0.0110; WT vs. Gαq/11 KO MEFs, t=5, *p*-value=0.0094; WT vs. Gαq/11 KO MEFs, t=15, *p*-value=0.0315. Autophagic vacuoles in WT and Gαq/11 KO MEFs were quantified using the Cyto-ID Autophagy detection Kit as in Suppl. Fig.1b. Cells were treated with chloroquine (CQ) (50µM) to analyze autophagic flux. Statistical significance was analyzed using two-sided unpaired *t*-test. For all *p*-values, *p<0.05, **p<0.01, ***p<0.001, ****p<0.0001. Representative histograms are shown: WT MEFs are represented in green and Gαq/11 KO MEFs in orange. d) Example of the gating strategy used in cytometry experiments. The starting cell population was gated for FSC-H/SSC-H in order to identify single cell population. This single cell population was analyzed for FL1-Cyto-ID histogram plot considering values $<10^1$ as negative staining cell populations and values $>10^1$ as positive staining cell population. WT MEFs negative and positive controls are shown. Source data are provided as a Source Data file.

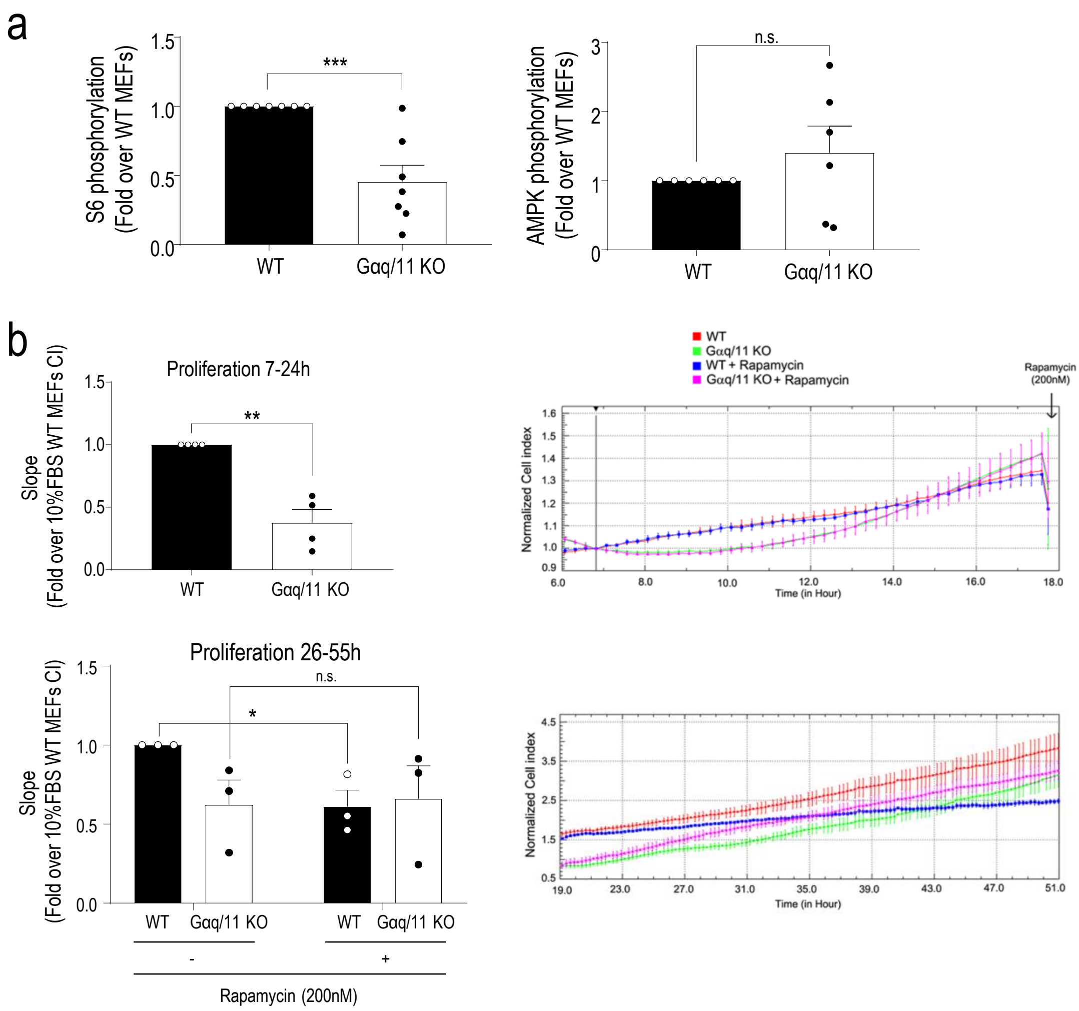

Supplementary Figure 5

Supplementary Fig.5. Gαq/11 KO MEFs display lower basal mTORC1 pathway activation status and proliferation rates compared to WT cells. a) Quantification of the basal phosphorylation status of S6 ribosomal protein (n=7) and AMPK (n=6) in WT and Gαq/11 KO MEFs. Data (mean ± SEM of the indicated independent experiments) were normalized with total levels of S6 ribosomal protein or AMPK, respectively, and expressed as fold-change over WT MEFs WT vs. Gαq/11 KO MEFs, S6 phosphorylation, *p*-value=0.0007; WT vs. Gαq/11 KO MEFs, AMPK phosphorylation, *p*-value=0.3238. b) WT and Gαq/11 KO MEFs were seeded into 96-well gold electrode sensor plates in 10% FBS. 18h after plating, cells were treated with rapamycin (200nM) or vehicle and maintained for an additional period of 32h. Monitoring of cell index changes with xCELLigence technology was performed every 15min. Cell index (CI) values were normalized 7h after plating (to exclude the adhesion phase). Cell growth was calculated as the slope (hours-1) of the cell index curve during a total of 51h. The 7-24h (n=4) period shows the first proliferation phase (WT vs. Gαq/11 KO MEFs, *p*-value=0.0010), and 26-55h (n=3) the phase after the addition of Rapamycin (200nM). WT(-Rapa) vs. WT(+Rapa) MEFs, *p*-value=0.0209; Gαq/11 KO(-Rapa) vs. Gαq/11 KO(+Rapa) MEFs, *p*-value=0.8942. Slope data was expressed as fold-induction CI values over WT MEFs not treated with rapamycin and are the mean ± SEM of the indicated independent experiments. Statistical significance was analyzed using two-sided unpaired *t*-test. For all *p*-values, *p<0.05, **p<0.01, ***p<0.001. Source data are provided as a Source Data file.

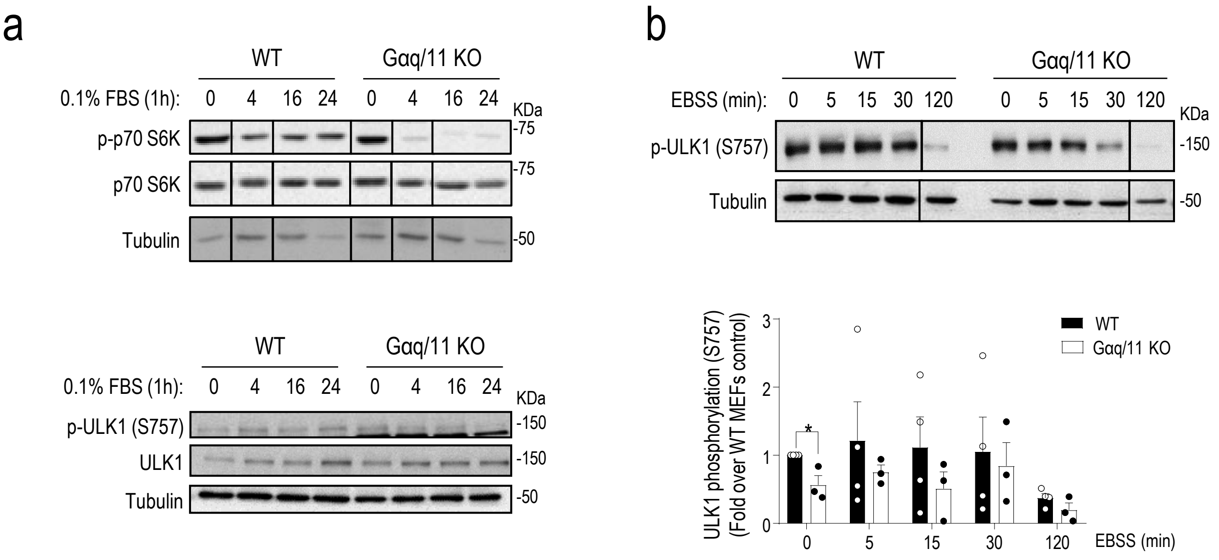

Supplementary Figure 6

Supplementary Fig.6. Cells lacking Gαq/11 display an altered mTORC1 activation status under different nutrient stress conditions. WT and Gαq/11 KO MEFs were maintained in presence or absence of serum (a) or amino acids (b) during the indicated times. Activation levels of the mTORC1 pathway were analyzed by assessing the phosphorylation status of the downstream targets of mTORC1 (p70 S6 kinase and ULK1-Ser757). Tubulin was used as loading control. A representative blot of 4 independent experiments is shown. ULK1 phosphorylation (S757) (mean ± SEM of n=4 (WT) and n=3 (Gαq/11 KO) independent experiments) was normalized using Tubulin. Statistical significance was analyzed using two-sided unpaired *t*-test. WT vs Gαq/11 KO MEFs, t=0, *p-value*=0.0133. Source data are provided as a Source Data file.

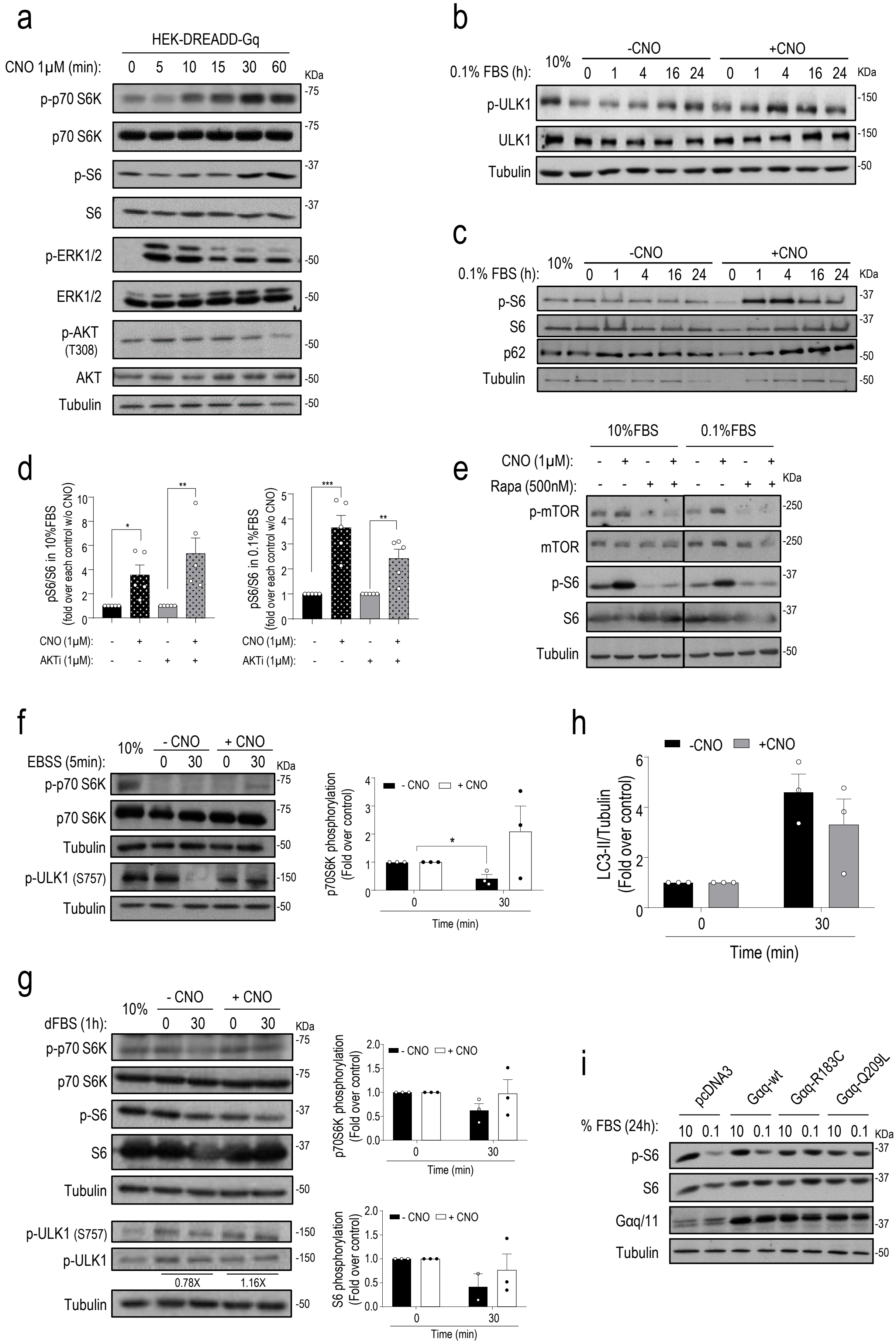

Supplementary Figure 7

Supplementary Fig.7. The specific activation of Gαq/11 modulates mTORC1/autophagy pathways. a) Characterization of the DREADD-Gq HEK-293 system. Cells were starved for 16h with serum-free medium and stimulated with CNO (1μM) for different times. The modulation of autophagy was analyzed by assessing the phosphorylation status of the downstream targets of mTORC1 (p70 S6 kinase and S6 ribosomal proteins). ERK1/2 and Akt (Thr308) phosphorylation were checked as positive controls for pathways known to be stimulated downstream of Gαq/11. A representative blot of 3 independent experiments is shown. b-c) Cells growing in 10% FBS were starved for 24h with 0.1% FBS medium and stimulated with CNO for different times as in Fig.3c. Lysates were analyzed for the activation of mTORC1 by assessing the phosphorylation status of downstream targets of mTORC1 (ULK1 (S757) and S6 ribosomal proteins) and for the autophagic marker p62. Total ULK1, total S6 ribosomal protein and tubulin were used as loading controls. Representative blots of 3 independent experiments are shown. d) Cells growing in 10% FBS or starved for 24h with 0.1% FBS medium were stimulated with CNO (1μM) or vehicle for 4h in the absence or presence of the AKTi-1/2 inhibitor (1μM). Phospho-S6 data (mean ± SEM of 5 independent experiments) were normalized using total S6 ribosomal protein. pS6/S6 in 10%FBS: -CNO vs. +CNO, *p*-value=0.0144; -CNO(+AKTi) vs. +CNO(+AKTi), *p*-value=0.0100. pS6/S6 in 0.1%FBS: -CNO vs. +CNO, *p*-value=0.0007; -CNO(+AKTi) vs. +CNO(+AKTi), *p*-value=0.0054. e) Cells growing in 10% FBS or starved for 24h with 0.1% FBS medium were stimulated with CNO (1μM) or vehicle for 4h in the absence or presence of the mTOR inhibitor rapamycin (500 μM) and mTOR and S6 phosphorylation status assessed with specific antibodies. Tubulin was used as loading control. Representative blots of 3 independent experiments are shown. f) Cells growing in 10% FBS were starved for 5min with EBSS and stimulated or not with CNO for 30min and mTORC1 pathway readouts analyzed as in previous figures. Phospho p70-S6K data (mean ± SEM of 3 independent experiments) were normalized using total p70-S6K protein. -CNO(t=0) vs. -CNO(t=30), *p*-value=0.0158. g) Cells growing in 10% FBS were starved for 1h with RPMI medium (without amino acids) supplemented with dialyzed serum (dFBS) (without amino acids), followed by stimulation with CNO for 30min. The modulation of the mTORC1 pathway was analyzed as in Fig.3e. Phospho-p70 S6K and pS6 ribosomal protein data (mean ± SEM of 3 independent experiments) were normalized using respective total protein levels. ULK1 (S757) phosphorylation fold modulation in response to CNO is indicated in the representative blot shown. h) Cells starved for 5min with EBSS were stimulated with CNO for 30min (see conditions in Fig.3e). Basal autophagy levels were checked by analyzing LC3-II protein levels by western blot. Data (mean ± SEM of 3 independent experiments) were normalized using tubulin as loading control and expressed as fold-change with respect to the control condition (t=0 min). i) CHO cells transfected with plasmid constructs encoding Gαq

wt, or the constitutively active Gq-R183C or Gq-Q209L mutants were starved with 0.1% FBS for 24h. A representative blot of 3 independent experiments is shown. In the indicated panels, statistical significance was analyzed using two-sided unpaired *t*-test. For all *p*-values, *p<0.05, **p<0.01, ***p<0.001. Source data are provided as a Source Data file.

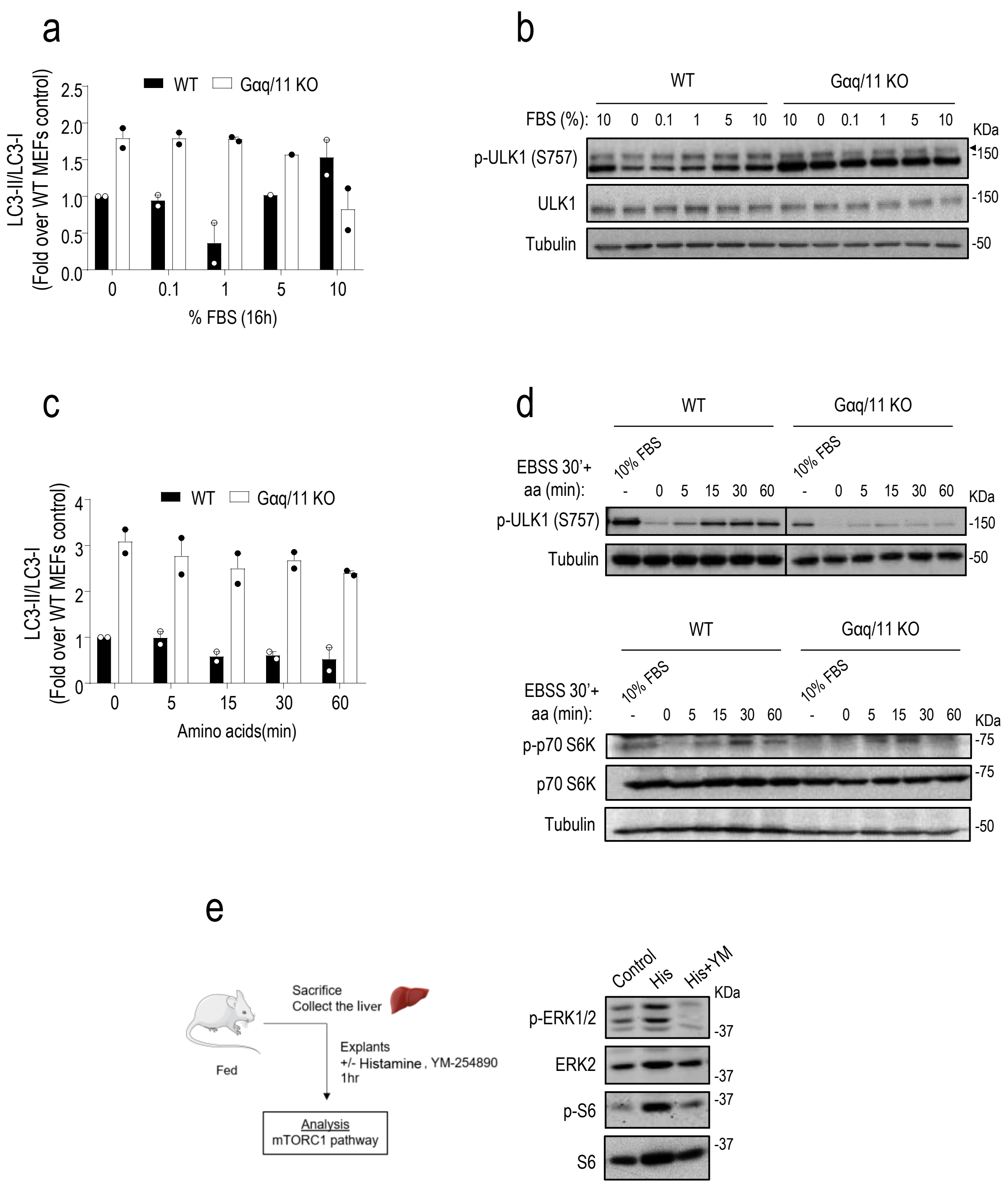

Supplementary Figure 8

Supplementary Fig.8. Gαq/11 is required to reactivate the mTORC1 pathway and thus inactivate autophagy in response to nutrient recovery. a) LC3-II/LC3-I ratio (n=2, except for 5%FBS condition, n=1) from the experiments depicted in Fig. 4a (mean ± SEM of the indicated independent experiments). b) WT and Gαq/11 KO MEFs were treated as in Fig. 4a and lysates analyzed for ULK1 phosphorylation (S757). Tubulin and total ULK1 were used as loading controls. Representative blots of 3 independent experiments are shown. c) LC3-II/LC3-I ratio (n=2) from the experiments depicted in Fig. 4b (mean ± SEM of the indicated independent experiments). d) WT and Gαq/11 KO MEFs were treated as in Fig. 4b and lysates analyzed for p70 S6K and ULK1 phosphorylation (S757). Tubulin and total p70-S6K protein were used as loading controls. Representative blots of 3 independent experiments are shown. e) ERK1/2 and mTORC1 pathway analysis in liver explants from fed mice subjected to Histamine (200μM) or YM-254890 (40μM) treatments for 1hr. The images used in this diagram are from Servier Medical Art (http://smart.servier.com/). Representative blots for the activation of ERK1/2 and ribosomal S6 protein are shown. (n=2 mice). Source data are provided as a Source Data file.

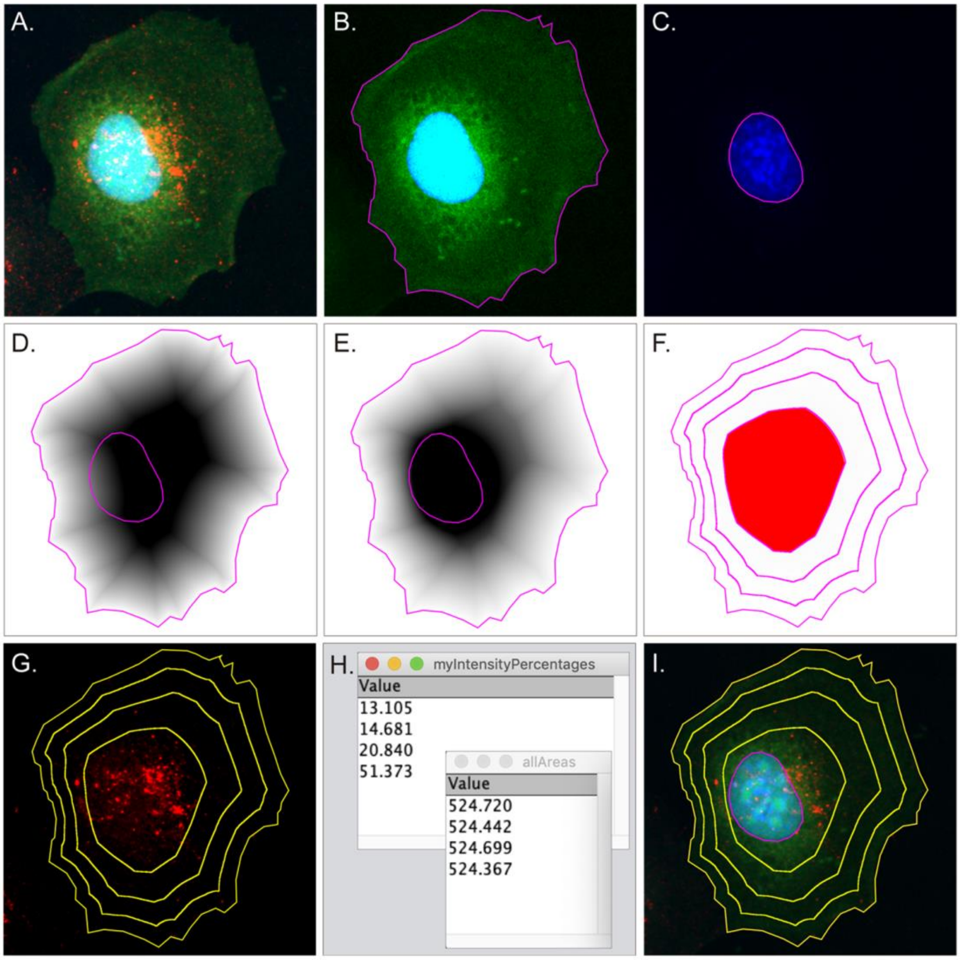

Supplementary Figure 9

Supplementary Fig.9. Signal intensity distribution from nucleus to cell periphery. An original Fiji macro has been developed that divides the cell into rings of equivalent area, which converge towards the nucleus center; the intensity density of the interest signal is then measured per ring and normalized to the total intensity density of the cell. In this manner, the intensity distribution of a target protein can be compared between cells of different shapes and sizes. The script is available at https://github.com/MolecularImagingPlatformIBMB/ringIntensityDistribution. We here used three-channel images [A] containing the signal of interest (red; lysosomal marker LAMP1), a cell tracer (green; anti-rabbit 488) and a nuclear marker (blue; Dapi). First of all, the cell boundary [B] and the nuclear boundary [C] were extracted from their corresponding channels, using respectively manual and automatic segmentation. Next, the provided script was used to: [D] create a distance map within the cell ROI; [E] correct the distance map values to make them converge at the centroid of the nuclear ROI; [F] threshold the distance map iteratively until the selected regions match the 75%, 50% and 25% of the total cell area, thus creating three rings plus a central region; [G] measure, on the channel containing the lysosomal signal, the fluorescence intensity density per ring, which is further normalized by the total cell intensity density [H]. [I] shows a reference snapshot of the same cell containing the three channels plus all the ROIs created.

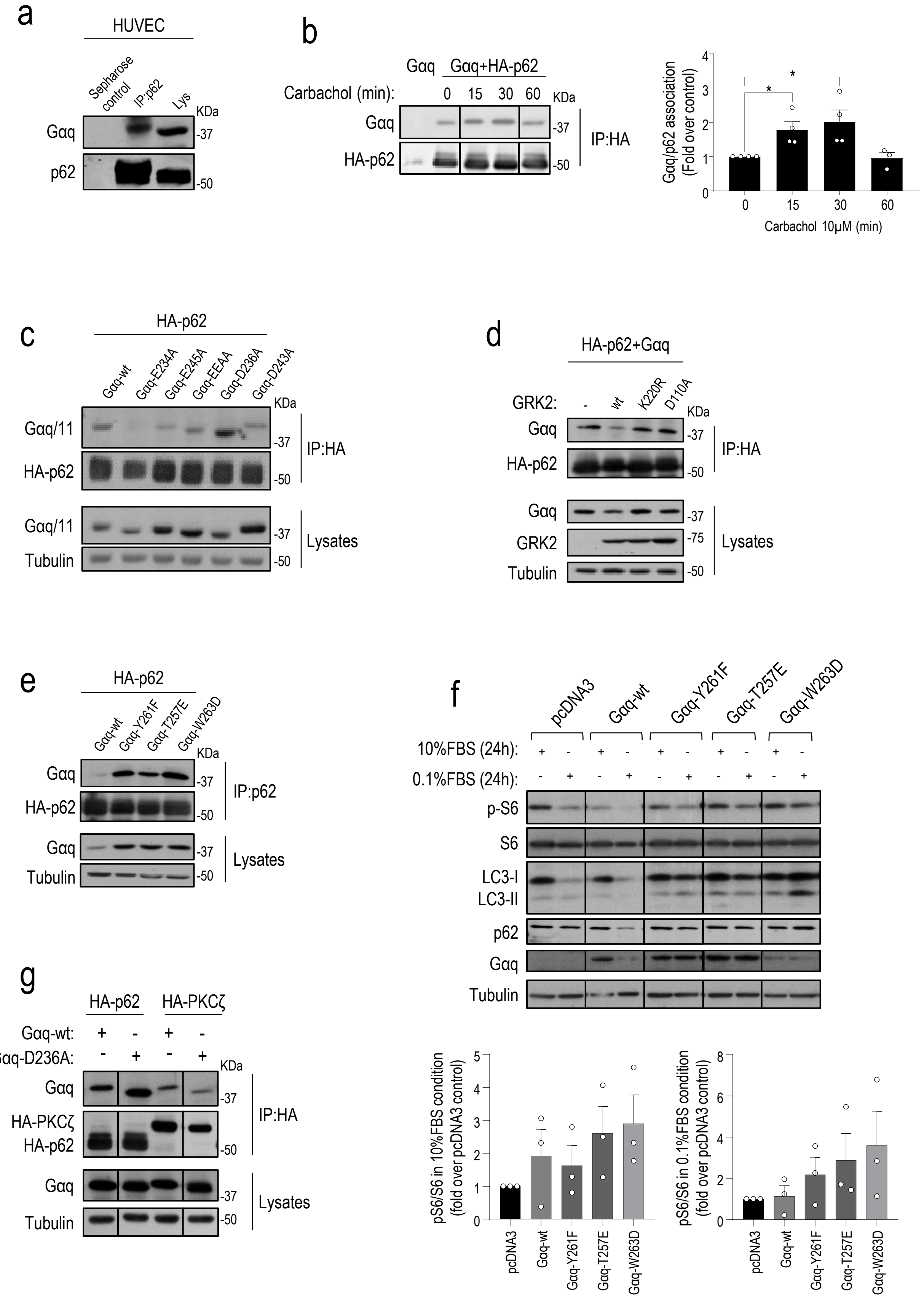

Supplementary Figure 10

Supplementary Fig.10. p62 displays features of a Gαq effector and mTORC1 and autophagy modulation by Gαq correlate with its ability to interact with p62. a) Endogenous Gq/p62 co-immunoprecipitation in HUVEC cells was performed under nutrient sufficiency conditions (10% FBS). Blots are representative of 2 independent experiments. b) CHO cells stably expressing the Gq-coupled muscarinic M3 receptor (CHO M3) were transfected with HA-p62 and Gαq constructs. 24 hours after transfection, cells were serum-starved for 2h and then stimulated with carbachol (10μM) for the indicated times. Data (mean ± SEM of n=4(t=0, t=15 and t=30) and n=3(t=60) independent experiments) were normalized with total HA-p62 and expressed as fold-induction of Gαq/p62 association in response to carbachol. Statistical significance was analyzed using two-sided unpaired *t*-test. For all *p*-values, *p<0.05. t=0 vs. t=15, *p*-value=0.0153; t=0 vs. t=30, *p*-value=0.0261. c) CHO cells were transiently transfected with the indicated combinations of HA-p62, Gαq-wt or Gαq mutants in the region involved in binding to PB1-domain-containing proteins (Gαq-E234A, Gαq-E245A, Gαq-EEAA., Gαq-D236A, Gαq-D243A) d) CHO cells were transfected with plasmids encoding HA-p62 and Gαq and the indicated combinations of GRK2 wild type, GRK2-K220R (kinase-inactive mutant) and GRK2-D110A (Gαq-interaction deficient mutant). Tubulin was used as loading control for lysates. Representative blots are shown. e) CHO cells were transfected with HA-p62 and combinations of plasmids encoding Gαq-wt or mutants deficient in association with GRK2 (Gαq-Y261F, Gαq-T257E, Gαq-W263D). In all cases (c-e), co-immunoprecipitation assays and expression controls in lysates were performed as described under Methods. Tubulin was used as loading control. Blots representative of 3 independent experiments are shown. f) HEK 293 DREADD Gαq cells growing in 10% FBS or starved for 24h with 0.1% FBS medium were transiently transfected with plasmids encoding Gαq-wt or mutants deficient in association with GRK2 (Gαq-Y261F, Gαq-T257E, Gαq-W263D) displaying increased association to p62, and lysates were analyzed for autophagic markers (LC3I/II and p62) and the phosphorylation status of S6 ribosomal protein. Tubulin was used as loading control. Phospho-S6 data (mean ± SEM of 3 independent experiments) were normalized using total S6 ribosomal protein and expressed as fold-change with respect to control cells transfect with pcDNA3 plasmid. g) Gαq associates with p62 in a PKCζ-independent way. Gαq-wt and Gαq-D236A were compared in their ability to interact with HA-p62 or HA-PKCζ in CHO cells transfected with the indicated combinations of plasmid constructs. Blots are representative of 3 independent experiments. Anti-HA immunoprecipitates were analyzed by western blot with specific antibodies as in previous figures to determine the relative presence of proteins in the complex and tubulin was used as loading control. Source data are provided as a Source Data file.

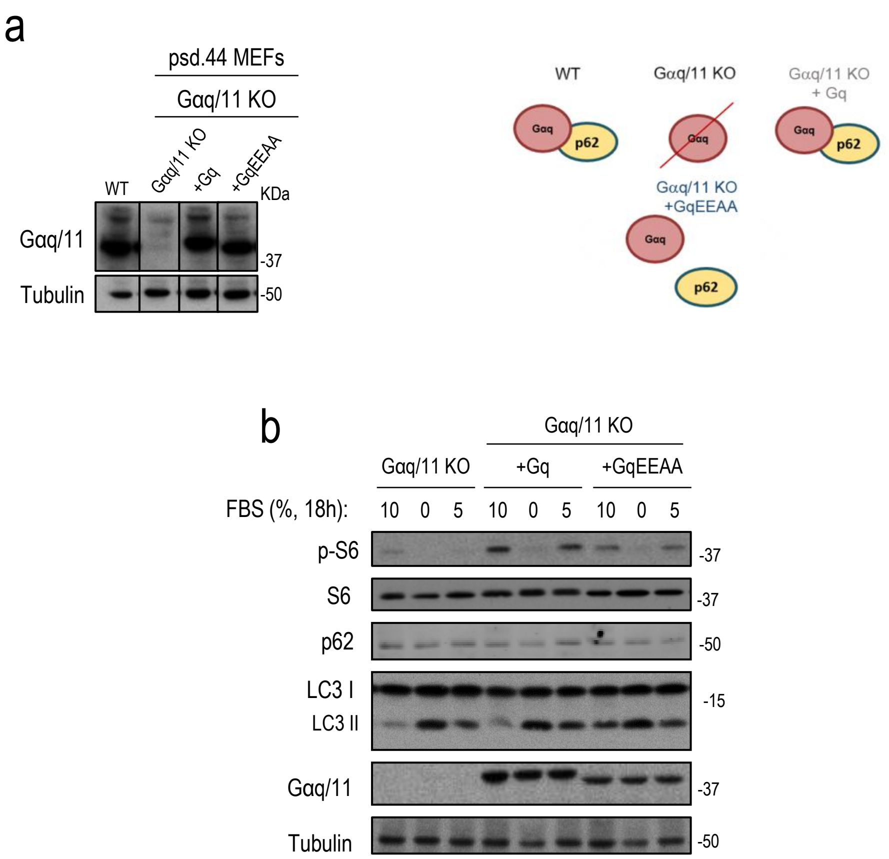

Supplementary Figure 11

Supplementary Fig.11. mTORC1 reactivation and autophagy modulation in response to serum recovery after starvation correlate with the ability of Gαq to interact with p62. a) A representative blot of Gαq/11 levels is shown in order to evaluate the rescue of Gαq protein expression in different MEF cell lines generated upon stable lentiviral expression of the indicated Gαq constructs with different ability to interact with p62 in the KO background. b) mTORC1 reactivation and autophagy modulation in response to serum recovery after starvation correlate with the ability of Gαq to interact with p62. The modulation of autophagy markers (LC3I/II and p62) and mTORC1 pathway (phosphorylation of S6 ribosomal protein) were analyzed by western blot in the different cell lines under serum starvation (0% FBS, for 18h) and in response to serum recovery (5% FBS, 18h). Tubulin was used as loading control. Blots are representative of 3 independent experiments.

Table 1. Primers

GqQ209L_FW	GAT GTA GGG GGC CTA AGG TCA GAG AGA
GqQ209L_REV	TCT CTC TGA CCT TAG GCC CCC TAC ATC
GqE234A_FW	CTA GTA GCG CTT AGT GCA TAT GAT CAA GTT CTC GTG G
GqE234A_REV	CCA CGA GAA CTT GAT CAT ATG CAC TAA GCG CTA CTA G
GqD236A_FW	GTA GCG CTT AGT GAA TAT GCG CAA GTT CTC GTG GAG TCA
GqD236A_REV	TGA CTC CAC GAG AAC TTG CGC ATA TTC ACT AAG CGC TAC
