# Supplementary material for: Gαq activation modulates autophagy by promoting mTORC1 signaling
Source: Nat Commun. 2021 Jul 27;12:4540. doi: 10.1038/s41467-021-24811-4 (PMC8316552; doi:10.1038/s41467-021-24811-4)
Supplement: Supplementary file 3 — Reporting Summary [file 41467_2021_24811_MOESM3_ESM.pdf]

nature research

Corresponding author(s): Catalina Ribas and Federico Mayor jr

Last updated by author(s): May 28, 2021

Reporting Summary

Nature Research wishes to improve the reproducibility of the work that we publish. This form provides structure for consistency and transparency in reporting. For further information on Nature Research policies, see our Editorial Policies and the Editorial Policy Checklist.

Statistics

For all statistical analyses, confirm that the following items are present in the figure legend, table legend, main text, or Methods section.

n/a	Confirmed	
☐	☒	The exact sample size (*n*) for each experimental group/condition, given as a discrete number and unit of measurement
☐	☒	A statement on whether measurements were taken from distinct samples or whether the same sample was measured repeatedly
☐	☒	The statistical test(s) used AND whether they are one- or two-sided<br>*Only common tests should be described solely by name; describe more complex techniques in the Methods section.*
☒	☐	A description of all covariates tested
☐	☒	A description of any assumptions or corrections, such as tests of normality and adjustment for multiple comparisons
☐	☒	A full description of the statistical parameters including central tendency (e.g. means) or other basic estimates (e.g. regression coefficient) AND variation (e.g. standard deviation) or associated estimates of uncertainty (e.g. confidence intervals)
☐	☒	For null hypothesis testing, the test statistic (e.g. *F*, *t*, *r*) with confidence intervals, effect sizes, degrees of freedom and *P* value noted<br>*Give P values as exact values whenever suitable.*
☒	☐	For Bayesian analysis, information on the choice of priors and Markov chain Monte Carlo settings
☒	☐	For hierarchical and complex designs, identification of the appropriate level for tests and full reporting of outcomes
☒	☐	Estimates of effect sizes (e.g. Cohen's *d*, Pearson's *r*), indicating how they were calculated

Our web collection on statistics for biologists contains articles on many of the points above.

Software and code

Policy information about availability of computer code

Data collection	Software used for data collection:<br>- xCELLigence system RTCA SP instrument 1.2.1 (Roche Applied Science) version for cell proliferation assays.<br>- Biorad GS-900 5.2 scanner or the Odyssey (Apllication Software 3.0 (Li-COR, Bioscience)) Infra-red Imaging System for collecting WB data<br>- BD FacsCalibur flow cytometer (BD-Bioscience) to collect flow cytometry data<br>- Sample images were acquired using a confocal laser microscope LSM710 (Zeiss) and 96-well plate images were captured with a high-content microscope (Operetta system, Perkin Elmer).
Data analysis	-Lysosome distribution was analyzed using an original ImageJ/Fiji 1.51n script developed to that purpose, available at https://github.com/MolecularImagingPlatformIBMB/ringIntensityDistribution/blob/master/ringIntensityDistribution.ijm, ImageJ / Fiji, from version 1.39 onwards uses a modification of the original "rolling ball" algorithm published in Stanley Sternberg's article, "Biomedical Image Processing", IEEE Computer, January 1983<br>-Immunoreactivity bands were quantified by laser densitometry with a Biorad GS-900 scanner and using the Bio-Rad provided Image Lab 5.2 (https://www.bio-rad.com) or by the software included in the Odyssey Infra-red Imaging System (Apllication Software 3.0 (Li-COR, Bioscience)).<br>-Flow cytometry data were analyzed with the FlowJo Software v7.6.5 and v10.0.8r1 (https://www.flowjo.com) and CellQuest Pro version 4.0 Software (BD-Bioscience)<br>-Image J software v1.53J (NIH) was used for general image analysis and manipulations. (https://imagej.nih.gov/ij/index.html).<br>-Excel (v1905) or GraphPad Prism v8 software (https://www.graphpad.com/) were used for data analysis

For manuscripts utilizing custom algorithms or software that are central to the research but not yet described in published literature, software must be made available to editors and reviewers. We strongly encourage code deposition in a community repository (e.g. GitHub). See the Nature Research guidelines for submitting code & software for further information.

Data

Policy information about availability of data

All manuscripts must include a data availability statement. This statement should provide the following information, where applicable:

- Accession codes, unique identifiers, or web links for publicly available datasets
- A list of figures that have associated raw data
- A description of any restrictions on data availability

Source data are provided with this paper: source data and uncropped gels are provided as source data files. List of Figures that have associated raw data are Figs 1 to 7 and Suppl Figs. 1 to 11. Other data that support the findings of this study are available from the corresponding authors upon reasonable request. An original Fiji macro has been developed and deposited in a Github repository (http://doi.org/10.5281/zenodo.4815136). No datasets were generated or analysed during the current study. All unique materials generated are readily available from the authors.

Field-specific reporting

Please select the one below that is the best fit for your research. If you are not sure, read the appropriate sections before making your selection.

☒ Life sciences ☐ Behavioural & social sciences ☐ Ecological, evolutionary & environmental sciences

For a reference copy of the document with all sections, see nature.com/documents/nr-reporting-summary-flat.pdf

Life sciences study design

All studies must disclose on these points even when the disclosure is negative.

Sample size	Sample size was chosen based on previous experience, on the type of experiment and on the anticipated variation according to previous experience from studies using related methods (Penela et al., Ebiomedicine 2019; Palacios et al., Int. J Cancer., 2020 and Cuervo et al., J Biol Chem., 1997; Klionsky et al., Autophagy. 2008).The number of animals used was stimated considering the minimum number to obtain statistical analysis to determine if there were differences between groups with a confidence level of 95% (p<0.05). We used at least 3-4 animals per group condition. Statistical analysis used in each figure are specified in figure legends.
Data exclusions	No data were excluded
Replication	All of the experiments were repeated for at least 3 times. Only experiments for Suppl Fig 8 a and c were done twice. All the attempts were successful.
Randomization	Animals were grouped based on their age or type of treatment. Randomization was not relevant for other experiments, as they were performed in cell lines
Blinding	Investigators were not blinded during animal experiments or in vitro treatment, data collection or analysis because the sample or animal names/number contained the information to allow the identification. For image analysis blinding of investigators was not necessary due to the utilization of investigator-independent unbiased methods of imaging data collection.

Reporting for specific materials, systems and methods

We require information from authors about some types of materials, experimental systems and methods used in many studies. Here, indicate whether each material, system or method listed is relevant to your study. If you are not sure if a list item applies to your research, read the appropriate section before selecting a response.

Materials & experimental systems

n/a	Involved in the study	
☐	☒	Antibodies
☐	☒	Eukaryotic cell lines
☒	☐	Palaeontology and archaeology
☐	☒	Animals and other organisms
☒	☐	Human research participants
☒	☐	Clinical data
☒	☐	Dual use research of concern

Methods

n/a	Involved in the study	
☒	☐	ChIP-seq
☐	☒	Flow cytometry
☒	☐	MRI-based neuroimaging

Antibodies

Antibodies used	Information regarding antibodies used in this study, including sources and catalog numbers, is provided in Methods: "Method details-SDS-PAGE and immunoblot analysis" subsection : Primary antibodies used in western blotting with dilutions were as follows: AKT (Cell signaling #9272; 1:1000), p-AKT (T308) (Cell signaling #9275; 1:1000), AMPKα (Cell signaling #2532; 1:1000), p-AMPK (T172) (Cell

signaling #2535; 1:1000), α-Tubulin (Santa Cruz #SC-53030; 1:1000), Erk1 (C-16, Santa Cruz #SC-16; 1:1000), Erk2 (C-14, Santa Cruz #SC-154; 1:1000), pERK1/2 (Thr202/204) (Cell Signaling, #9101; 1:1000), GAPDH (Abcam #ab8245; 1:5000), GFP (Santa Cruz #SC-9996; 1:1000), Gq/11 (C19, Santa Cruz #SC-392; 1:1000), Gq (E-17, Santa Cruz #SC-393; 1:1000), GRK2 (c-15, Santa Cruz #SC-562; 1:1000), HA (F-7, Santa Cruz #SC-7392; 1:1000), LAMP1 (1D4B, Hybridoma Bank; 1:3000), control IgG (normal Rabbit IgG sc-2027, Santa Cruz), LC3B (Cell signaling #2775; 1:1000), LC3B (NB100-2220, Novus Biologicals) mTOR (7C10, Cell signaling #2983; 1:1000), p62 (Progen, GP62; 1:1000), p62 (BML-PW9860, Enzo; 1:1000) p70-S6K (Cell signaling #2708; 1:1000), p-p70-S6K (Thr389) (Cell signaling #9205; 1:1000), Raptor (24C12) (Cell signaling, #2280; 1:1000), S6 ribosomal protein (Cell signaling #2217; 1:2000), p-S6 ribosomal protein (Ser240/244) (Cell signaling #2215; 1:1000), Ubiquitin (Sigma Aldrich #U5379; 1:50), ULK1 (D8H5, Cell signaling #8054; 1:1000), p-ULK1 (Ser757) (Cell signaling #6888; 1:1000), IRDyeTM 800 CW, donkey anti-guinea pig Igg, conjugated to infra-red dyes (LI-COR), Alexa 555, goat anti-rat IgG (ThermoFisher), Alexa 488, goat anti-mouse IgG1 ThermoFisher), Secondary antibodies coupled to HRP (Nordic Immunology)

Validation

All the antibodies used in this study are commercially available and validated on the manufacturer´s website:
-anti-AKT: https://www.cellsignal.com/products/primary-antibodies/akt-antibody/9272
-anti-p-AKT (S473): https://www.cellsignal.com/products/primary-antibodies/phospho-akt-ser473-d9e-xp-rabbit-mab/4060
-anti-p-AKT (T308): https://www.cellsignal.com/products/primary-antibodies/phospho-akt-thr308-antibody/9275
-anti-AMPKα: https://www.cellsignal.com/products/primary-antibodies/ampka-antibody/2532
-anti-p-AMPK (T172): https://www.cellsignal.com/products/primary-antibodies/phospho-ampka-thr172-40h9-rabbit-mab/2535
-anti-Tubulin: https://datasheets.scbt.com/sc-53030.pdf (has been discontinued)
-anti-Erk1: https://www.scbt.com/es/p/erk-1-antibody-c-16. ERK 1 (C-16) has been discontinued and replaced by ERK 1 (G-8): sc-271269.
-anti-pERK1/2 (Thr202/204): https://www.cellsignal.com/products/primary-antibodies/phospho-p44-42-mapk-erk1-2-thr202-tyr204-antibody/9101
-anti-GAPDH: https://www.abcam.com/gapdh-antibody-loading-control-ab9485.html
-anti-GFP: https://www.scbt.com/es/p/gfp-antibody-b-2
-anti-Gq/11: https://www.scbt.com/es/p/galpha-q-11-antibody-c-19. Gα q/11 (C-19) has been discontinued and replaced by Gα q/11/14 (G-7): sc-365906
-anti-Gq: https://www.scbt.com/es/p/galpha-q-antibody-e-17. Gα q (E-17) has been discontinued and replaced by Gα q/11/14 (G-7): sc-365906
-anti-GRK2: https://www.scbt.com/es/p/grk-2-antibody-c-15. GRK 2 (C-15) has been discontinued and replaced by GRK 2 (C-9): sc-13143
-anti-HA: https://www.scbt.com/es/p/ha-probe-antibody-f-7
-anti-mTOR: https://www.cellsignal.com/products/primary-antibodies/mtor-7c10-rabbit-mab/2983
-anti-LAMP1: https://dshb.biology.uiowa.edu/1D4B
-anti-LC3B: https://www.cellsignal.com/products/primary-antibodies/lc3b-antibody/2775
-anti-LC3B: https://www.novusbio.com/products/lc3b-antibody_nb100-2220
-anti-p62: https://www.progen.com/positive-western-blot-control-anti-p62-sqstm1-antibody.html?_ga=2.74434898.1783888057.1548168872-220732906.1548168872
-anti-p70-S6K: https://www.cellsignal.com/products/primary-antibodies/p70-s6-kinase-49d7-rabbit-mab/2708
-anti-p-p70-S6K: https://www.cellsignal.com/products/primary-antibodies/phospho-p70-s6-kinase-thr389-antibody/9205
-anti-Raptor: https://www.cellsignal.com/products/primary-antibodies/raptor-24c12-rabbit-mab/2280
-anti-Ubiquitin: https://www.sigmaaldrich.com/catalog/search?term=U5379&interface=All&N=0&mode=match%20partialmax&lang=es&region=global&focus=product
-anti-S6 ribosomal protein: https://www.cellsignal.com/products/primary-antibodies/s6-ribosomal-protein-5g10-rabbit-mab/2217
-anti-p-S6 ribosomal protein: https://www.cellsignal.com/products/primary-antibodies/phospho-s6-ribosomal-protein-ser240-244-antibody/2215
-anti-ULK1: https://www.cellsignal.com/products/primary-antibodies/ulk1-d8h5-rabbit-mab/8054
-anti-p-ULK1 (Ser757): https://www.cellsignal.com/products/primary-antibodies/phospho-ulk1-ser757-antibody/6888
-Secondary antibody coupled to HRP GAR/ gG: https://www.nordicmubio.com/products/goat-anti-rabbit-igg-heavy-and-light-chains-conjugated-with-horseradish-peroxidase/GAR_slash_IgG(H_plus_L)_slash_PO
-Secondary antibody GAM/IgG: https://www.nordicmubio.com/products/goat-anti-mouse-igg1-igg2a-igg2b-igg3-heavy-and-light-chains-conjugated-with-horseradish-peroxidase/GAM_slash_IgG(H_plus_L)_slash_PO
-IRDyeTM 800 CW donkey-anti-guinea-pig IgG Secondary Antibody : https://www.licor.com/bio/reagents/irdye-800cw-donkey-anti-guinea-pig-igg-secondary-antibody
-IRDye 800CW Donkey anti-Mouse IgG Secondary Antibody: https://www.licor.com/bio/reagents/irdye-800cw-donkey-anti-mouse-igg-secondary-antibody
-IRDye 680LT Donkey anti-Mouse IgG Secondary Antibody: https://www.licor.com/bio/reagents/irdye-680lt-donkey-anti-mouse-igg-secondary-antibody
-IRDye 800CW Goat anti-Rabbit IgG Secondary Antibody: https://www.licor.com/bio/reagents/irdye-800cw-goat-anti-rabbit-igg-secondary-antibody
-IRDye 680LT Goat anti-Rabbit IgG Secondary Antibody: https://www.licor.com/bio/reagents/irdye-680lt-goat-anti-rabbit-igg-secondary-antibody

Eukaryotic cell lines

Policy information about cell lines

Cell line source(s)

Cell sources have been provided in Methods "Experimental model and subject details" subsection: Gαq/11 WT and Gαq/11 KO MEFs were a kind gift from Dr. S. Offermanns (Max-Planck-Institute for Heart and Lung Research, Germany). Atg5 WT and Atg5 KO MEFs were provided by Dr. N. Mizushima (University of Tokio, Tokio, Japan) and DREADD-Gq-HEK293 cells (human female) by Dr. Silvio Gutkind (University of San Diego, California, USA) (Pei et al. 2008). CHO cells overexpressing the

	muscarinic M3 acetylcholine receptor (CHO-M3) (mice female) were a kind gift from Dr. A.B. Tobin (University of Glasgow, UK).
Authentication	The cell lines used have been authenticated by STR profiling or Barcode analysis.
Mycoplasma contamination	All cell lines were tested negative for mycoplasma contamination
Commonly misidentified lines (See ICLAC register)	No commonly misidentified cell lines were used in this study,

Animals and other organisms

Policy information about studies involving animals; ARRIVE guidelines recommended for reporting animal research

Laboratory animals	Rats<br>Adult male Wistar rats (200-250 g/ 8 weeks) fed or starved (4-48 hours) were used under an institutional approved animal study protocol. All rats were housed under pathogen-free conditions and handled according to protocols approved by the Institutional Animal Care and Use Committee of Albert Einstein College of Medicine (New York, USA).<br><br>Mice<br>Adult 8–12 weeks male inducible endothelium-specific Gαq/Gα11-deficient mice (Tie2-CreERT2; Gnaq f/f; Gna11 −/− [EC-q/11-KO] (Offermanns et al., 1997, 1998). Mice were housed under a 12-h light–dark cycle with free access to food and water and under specific pathogen–free conditions at 20-24 ºC with a humidity of 55 % ± 10%. All animal experimentation procedures conformed to the European Guidelines for the Care and Use of Laboratory Animals (Directive 86/609) and were approved by the Ethical Committees for Animal Experimentation of our University and the Comunidad Autónoma de Madrid, Spain.
Wild animals	No wild animals were used in the study
Field-collected samples	No field collected samples were used in the study.
Ethics oversight	All rats were housed under pathogen-free conditions and handled according to protocols approved by the Institutional Animal Care and Use Committee of Albert Einstein College of Medicine (New York, USA).<br><br>All animal experimentation procedures conformed to the European Guidelines for the Care and Use of Laboratory Animals (Directive 86/609) and were approved by the Ethical Committees for Animal Experimentation of our University and the Comunidad Autónoma de Madrid, Spain.

Note that full information on the approval of the study protocol must also be provided in the manuscript.

Flow Cytometry

Plots

Confirm that:

☒ The axis labels state the marker and fluorochrome used (e.g. CD4-FITC).

☒ The axis scales are clearly visible. Include numbers along axes only for bottom left plot of group (a 'group' is an analysis of identical markers).

☐ All plots are contour plots with outliers or pseudocolor plots.

☐ A numerical value for number of cells or percentage (with statistics) is provided.

Methodology

Sample preparation	To quantitatively measure apoptosis, the PE Annexin V Apoptosis Detection kit I (BD Bioscience) was utilized. Gαq/11 WT and Gαq/11 KO MEFs at 90% confluency were starved during 24h with 0.1% FBS after which cells were re-suspended in Annexin V-binding buffer (0.1M Hepes/NaOH (pH 7.4), 1.4M NaCl, 25mM CaCl2).
Instrument	Samples were analyzed by flow cytometry on a BD FacsCalibur flow cytometer (BD-Bioscience)
Software	CellQuest Software (BD-Bioscience) and FlowJo Software
Cell population abundance	Live cells (85-90%) were detected by Hoesch
Gating strategy	The starting cell population was gated for FSC-H/SCC-H in order to identify single cell population. This single cell population was analized for FL1-Cyto-ID histogram plot considering values under 10^1 as negative staining cell populations and values over 10^1 as positive staining cell population. See Suppl Fig 4d

☒ Tick this box to confirm that a figure exemplifying the gating strategy is provided in the Supplementary Information.
