# Supplementary material for: Gαq activation modulates autophagy by promoting mTORC1 signaling
Source: Nat Commun. 2021 Jul 27;12:4540. doi: 10.1038/s41467-021-24811-4 (PMC8316552; doi:10.1038/s41467-021-24811-4)
Supplement: Supplementary file 4 — Source data [file 41467_2021_24811_MOESM4_ESM.zip › Uncropped blots-NCOMMS-20-15236B.pdf]

Figure 1a

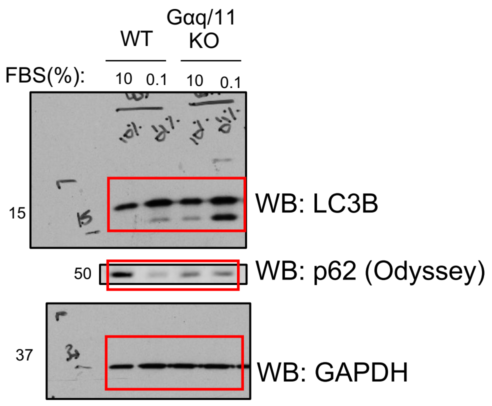

Figure 1b

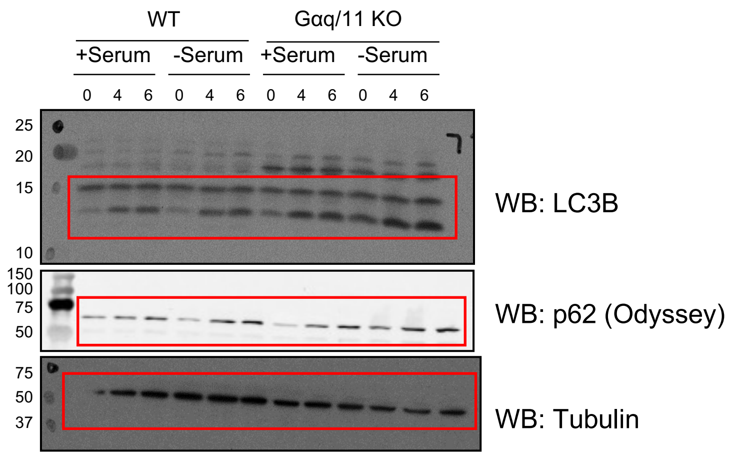

Figure 1h

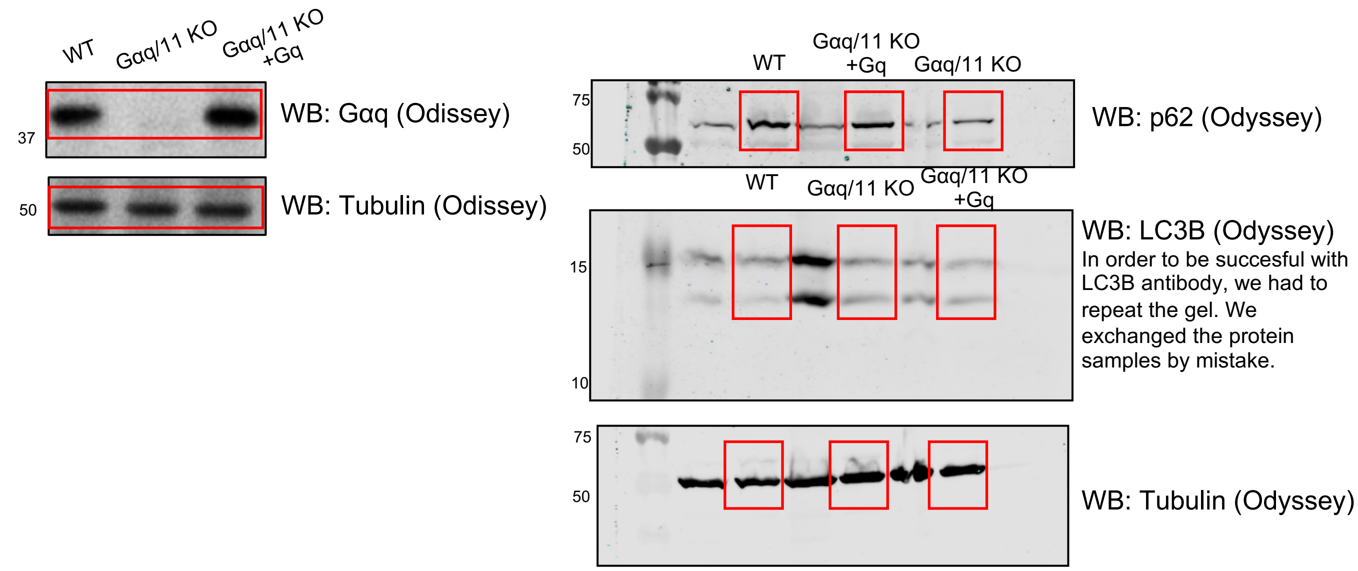

Figure 1i

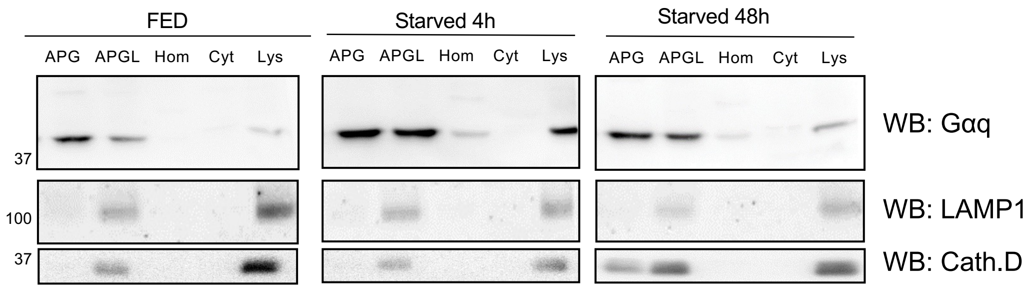

Figure 1

Figure 2a

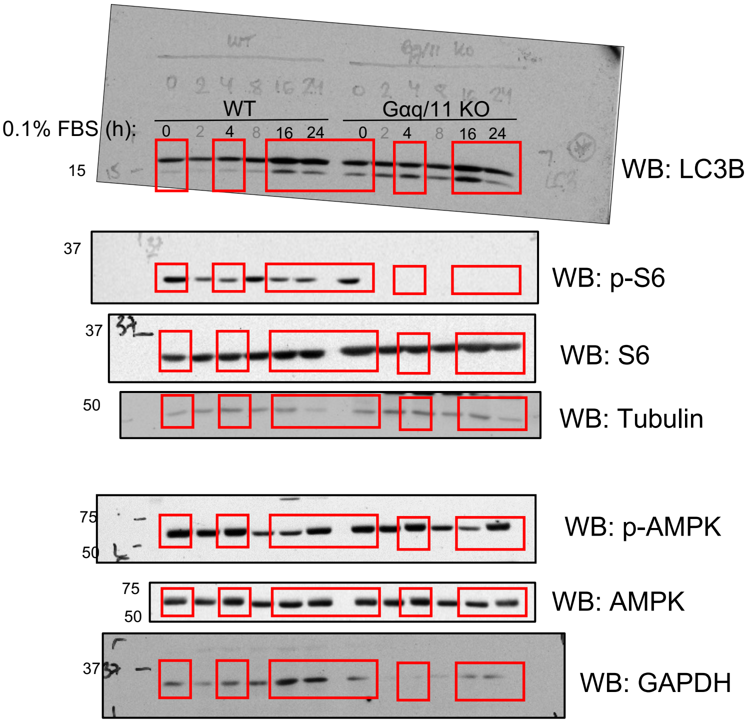

Figure 2b

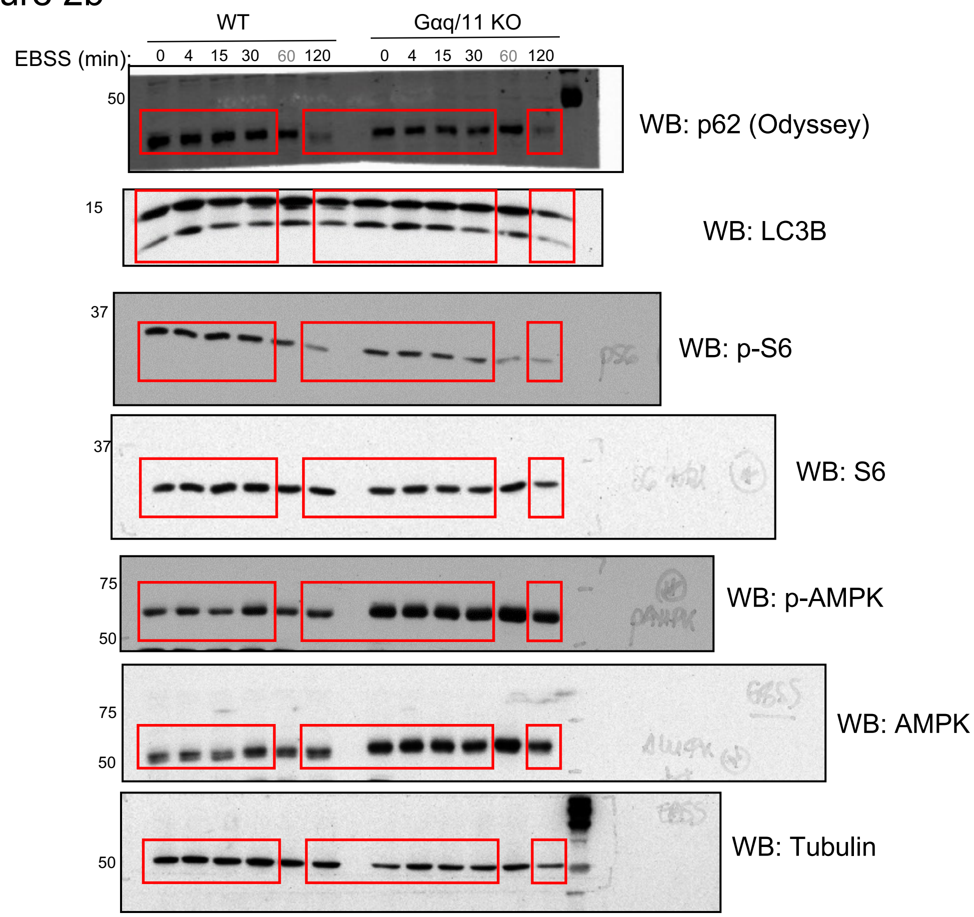

Figure 2

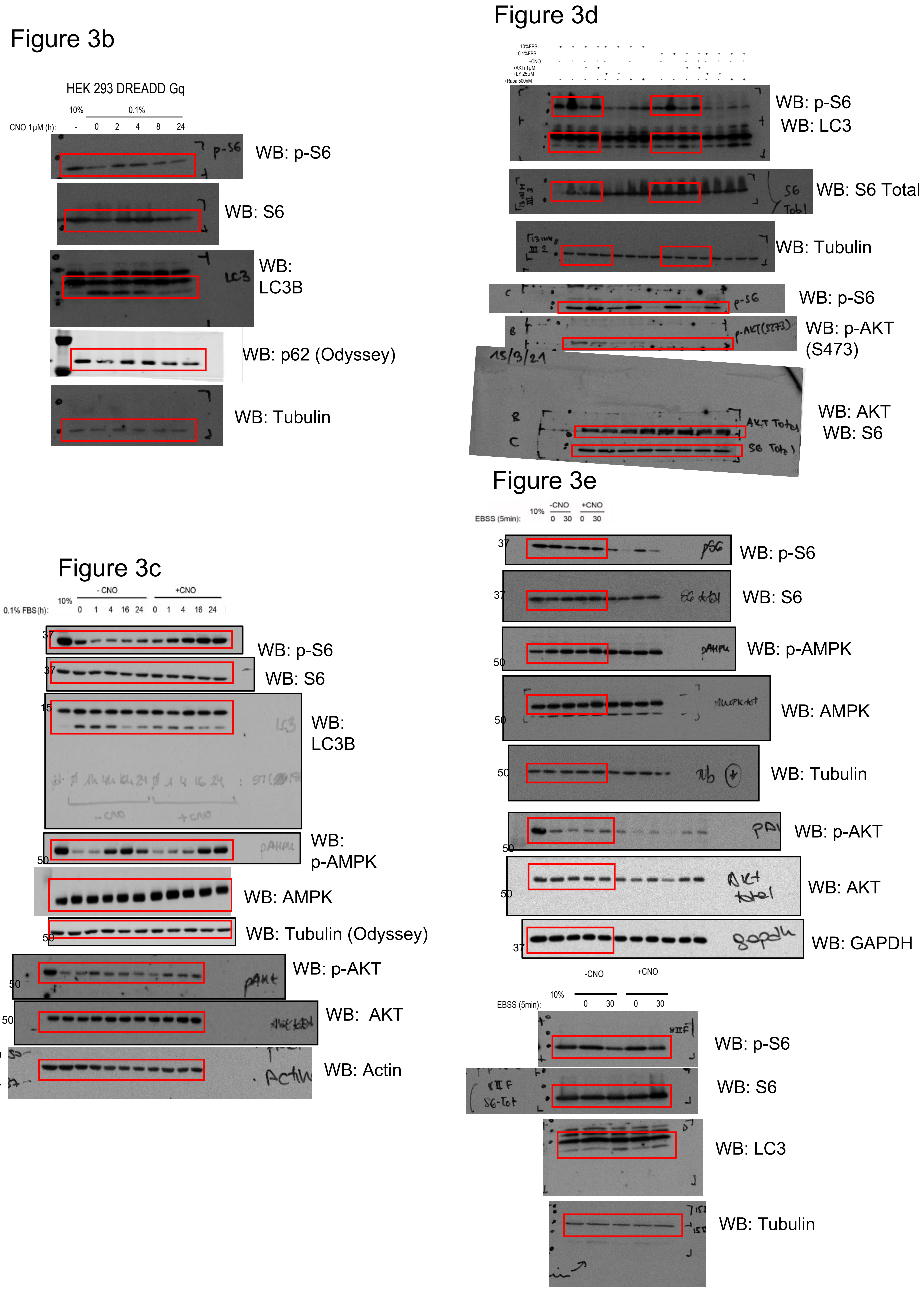
Figure 3b
HEK 293 DREADD Gq
10%
0.1%
CNO 1μM (h): - 0 2 4 8 24
WB: p-S6
WB: S6
WB: LC3B
WB: p62 (Odyssey)
WB: Tubulin
Figure 3c
- CNO
+CNO
10%
0.1% FBS (h): 0 1 4 16 24 0 1 4 16 24
WB: p-S6
WB: S6
WB: LC3B
WB: p-AMPK
WB: AMPK
WB: Tubulin (Odyssey)
WB: p-AKT
WB: AKT
WB: Actin
Figure 3d
10%FBS
0.1%FBS
+CNO
+AKTi 1μM
+LY 25μM
+Rapa 500nM
WB: p-S6
WB: LC3
WB: S6 Total
WB: Tubulin
WB: p-S6
WB: p-AKT (S473)
WB: AKT
WB: S6
Figure 3e
-CNO
+CNO
10%
EBSS (5min): 0 30 0 30
WB: p-S6
WB: S6
WB: p-AMPK
WB: AMPK
WB: Tubulin
WB: p-AKT
WB: AKT
WB: GAPDH
-CNO
+CNO
10%
EBSS (5min): 0 30 0 30
WB: p-S6
WB: S6
WB: LC3
WB: Tubulin

Figure 3

Figure 4a

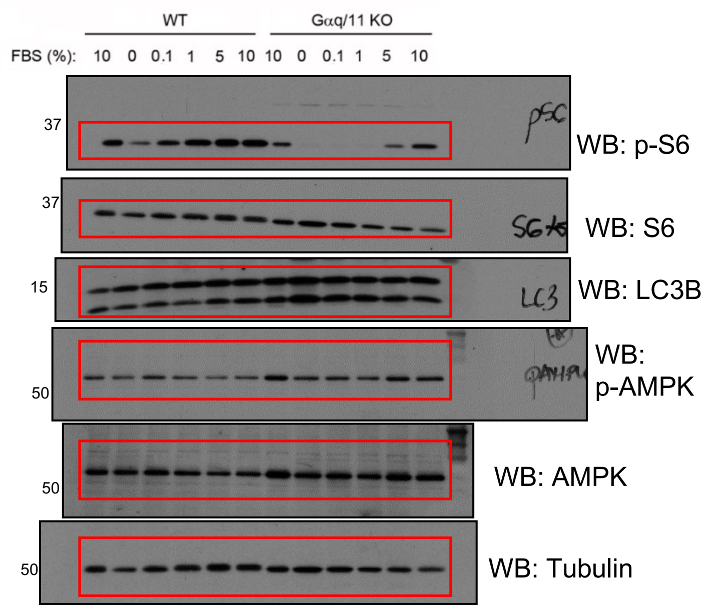

Figure 4b

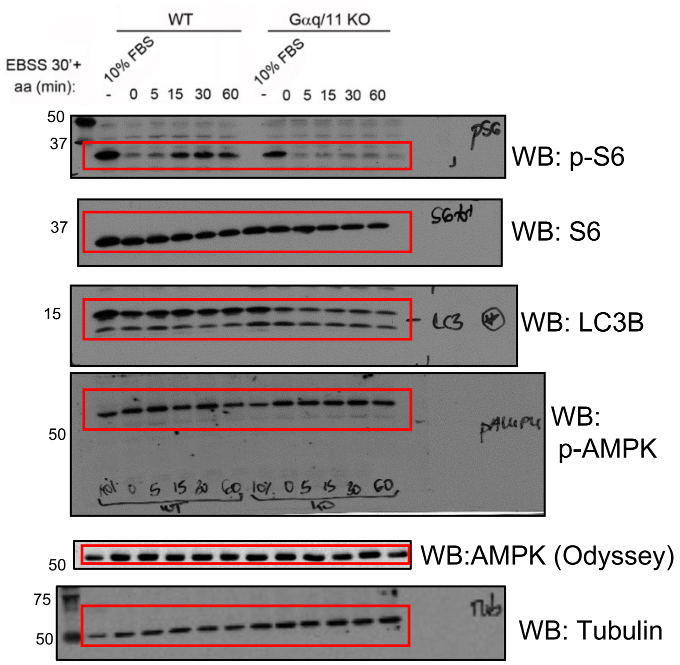

Figure 4d

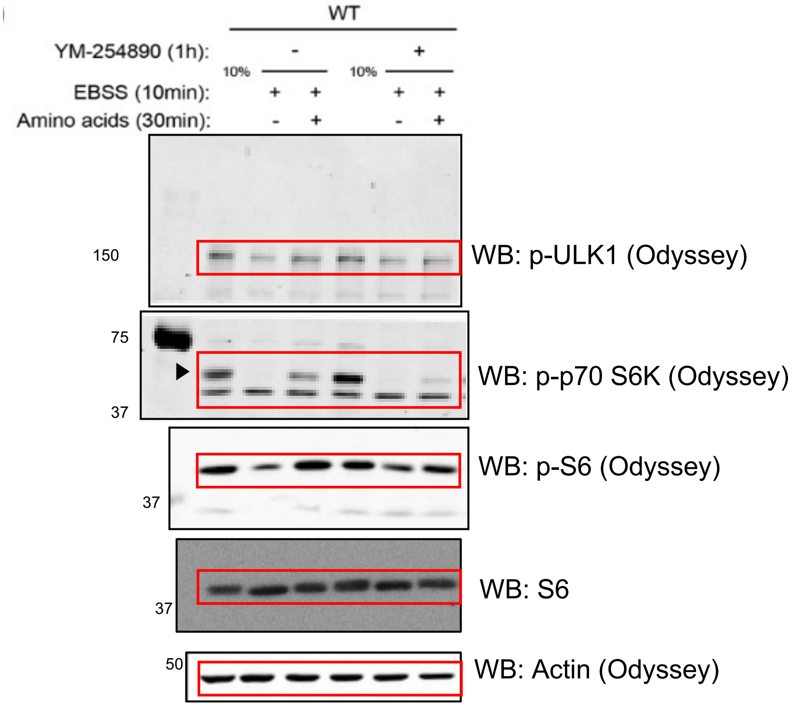

Figure 4f

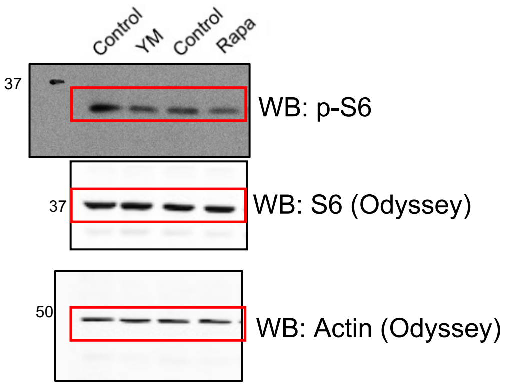

Figure 4

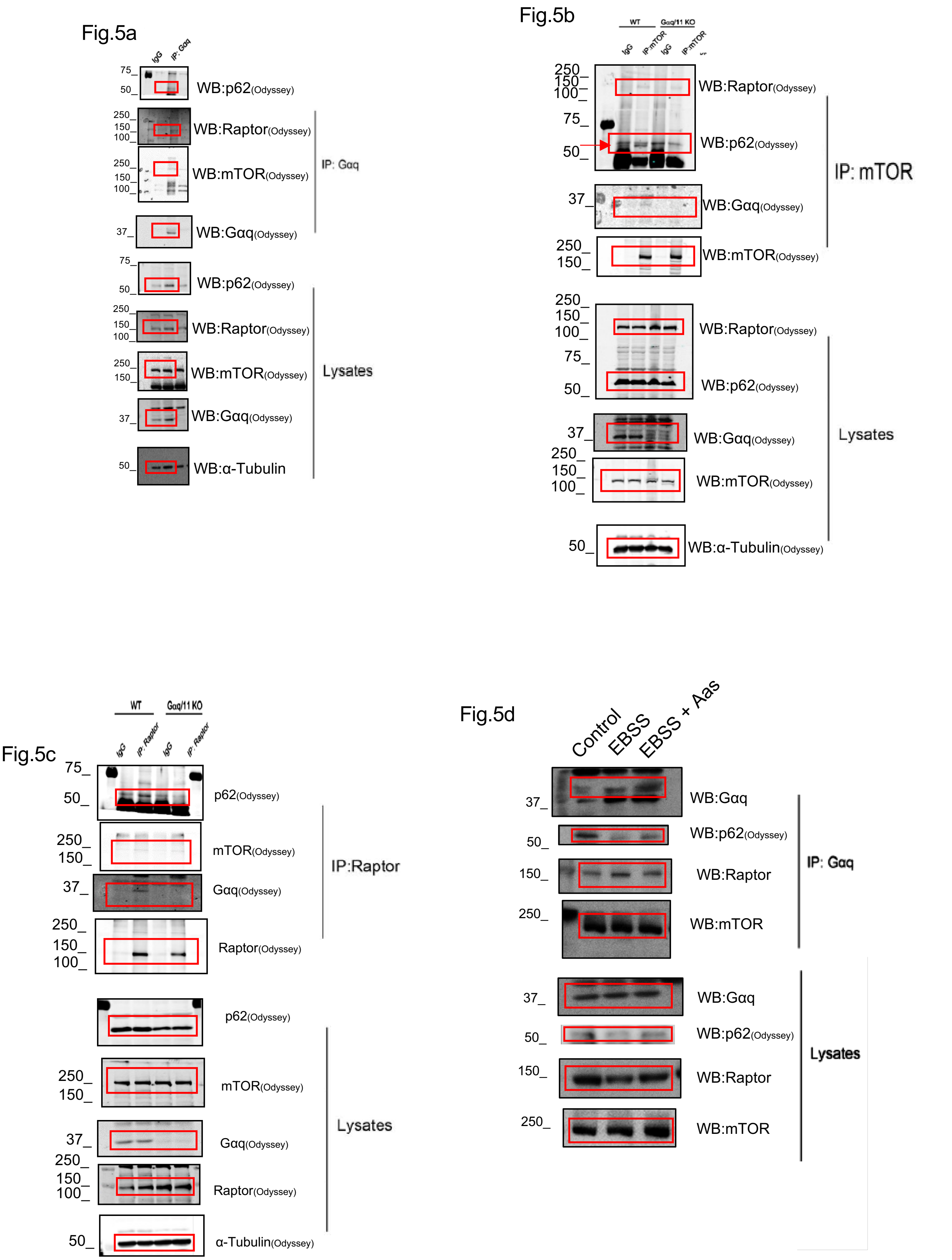
Fig.5a
IgG
IP: Gαq
75_
50_
WB:p62(Odyssey)
250_
150_
100_
WB:Raptor(Odyssey)
WB:mTOR(Odyssey)
37_
WB:Gαq(Odyssey)
IP: Gαq
WB:p62(Odyssey)
WB:Raptor(Odyssey)
WB:mTOR(Odyssey)
WB:Gαq(Odyssey)
WB:α-Tubulin
Lysates
Fig.5b
WT
Gαq/11 KO
IgG
IP:mTOR
IgG
IP:mTOR
250_
150_
100_
75_
50_
WB:Raptor(Odyssey)
WB:p62(Odyssey)
37_
WB:Gαq(Odyssey)
WB:mTOR(Odyssey)
IP: mTOR
WB:Raptor(Odyssey)
WB:p62(Odyssey)
WB:Gαq(Odyssey)
WB:mTOR(Odyssey)
WB:α-Tubulin(Odyssey)
Lysates
Fig.5c
WT
Gαq/11 KO
IgG
IP: Raptor
IgG
IP: Raptor
p62(Odyssey)
mTOR(Odyssey)
Gαq(Odyssey)
Raptor(Odyssey)
IP:Raptor
p62(Odyssey)
mTOR(Odyssey)
Gαq(Odyssey)
Raptor(Odyssey)
α-Tubulin(Odyssey)
Lysates
Fig.5d
Control
EBSS
EBSS + Aas
WB:Gαq
WB:p62(Odyssey)
WB:Raptor
WB:mTOR
IP: Gαq
WB:Gαq
WB:p62(Odyssey)
WB:Raptor
WB:mTOR
Lysates

Figure 5

Figure 6a

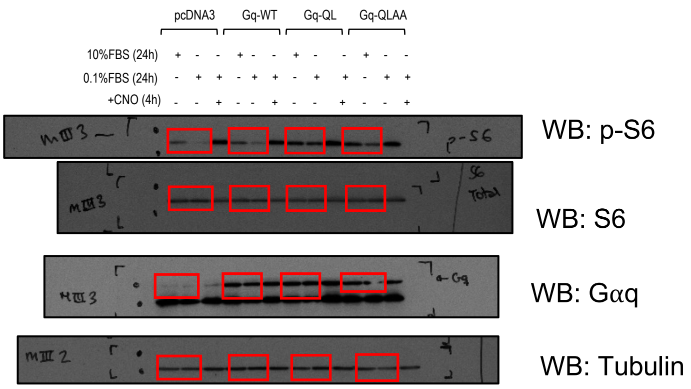

Figure 6b

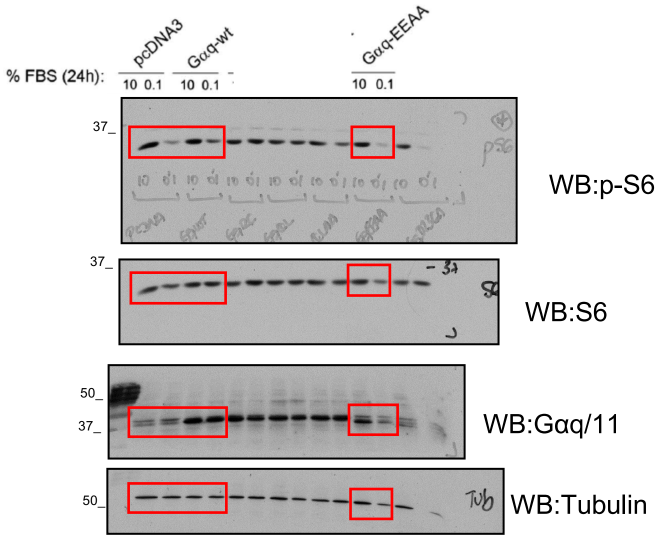

Figure 6c

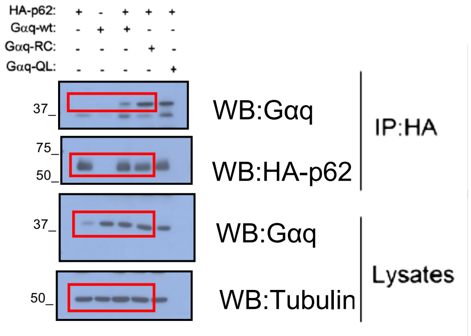

Figure 6d

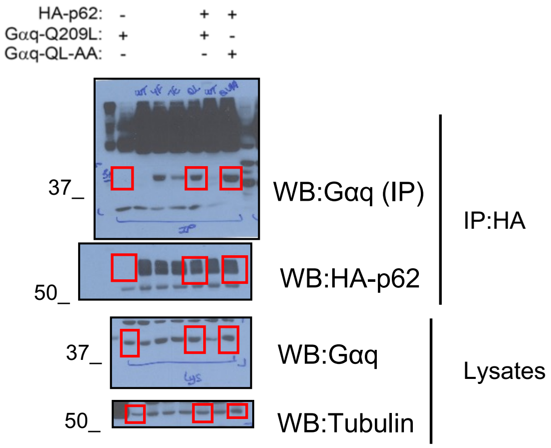

Figure 6e

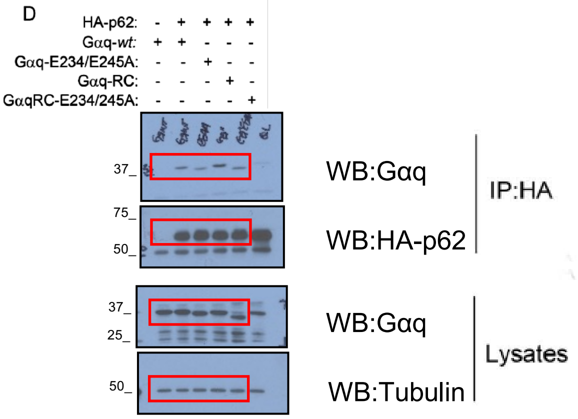

Figure 6f

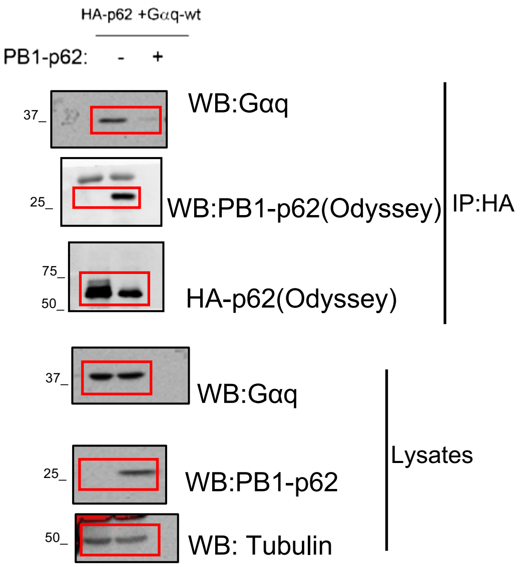

Figure 6

Figure 7a

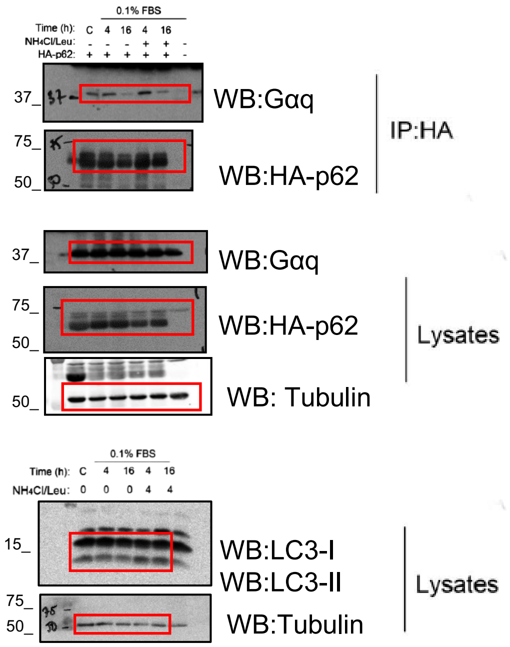

Figure 7b

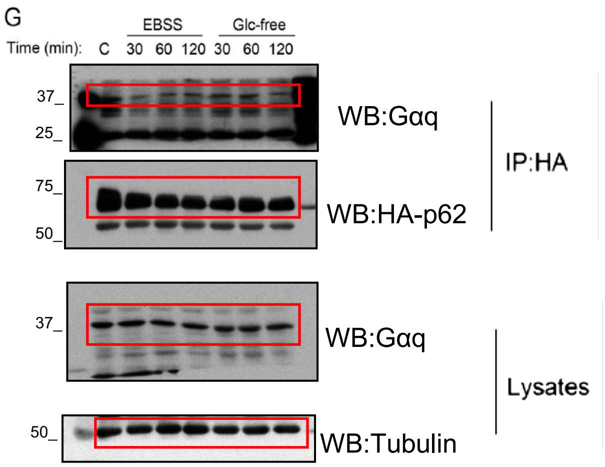

Figure 7d

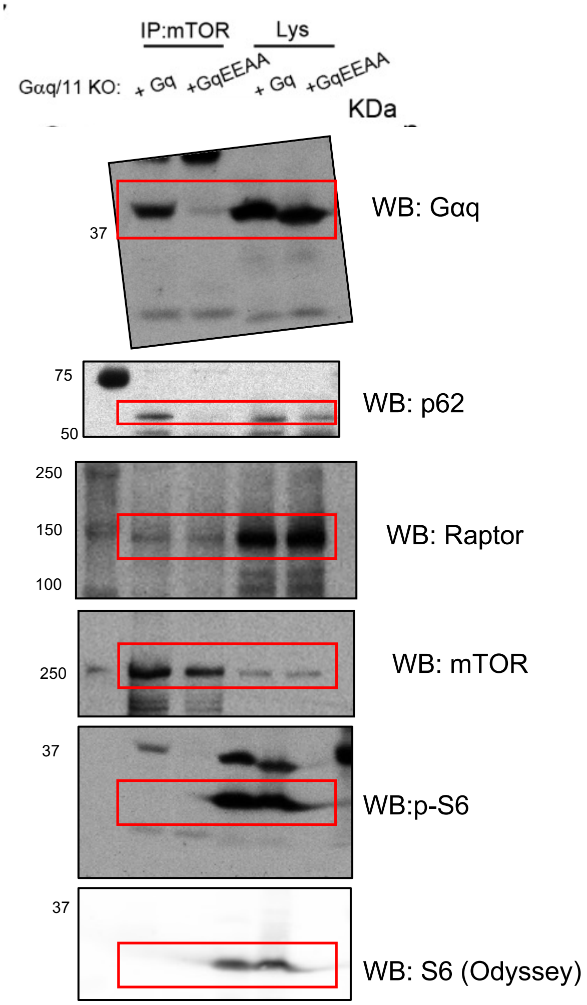

Figure 7e

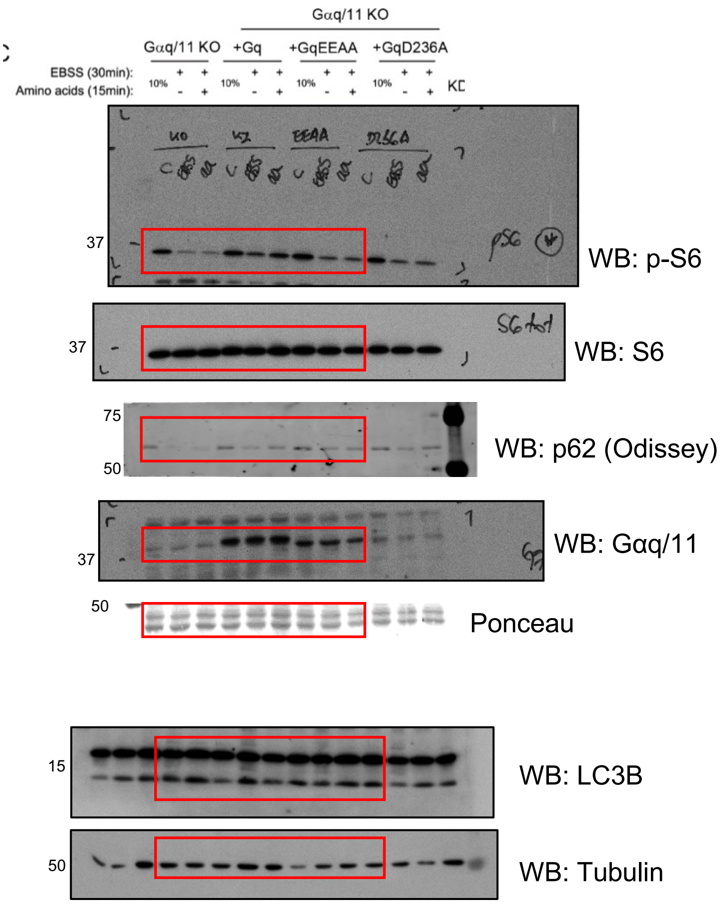

Figure 7

Supp Fig.1d

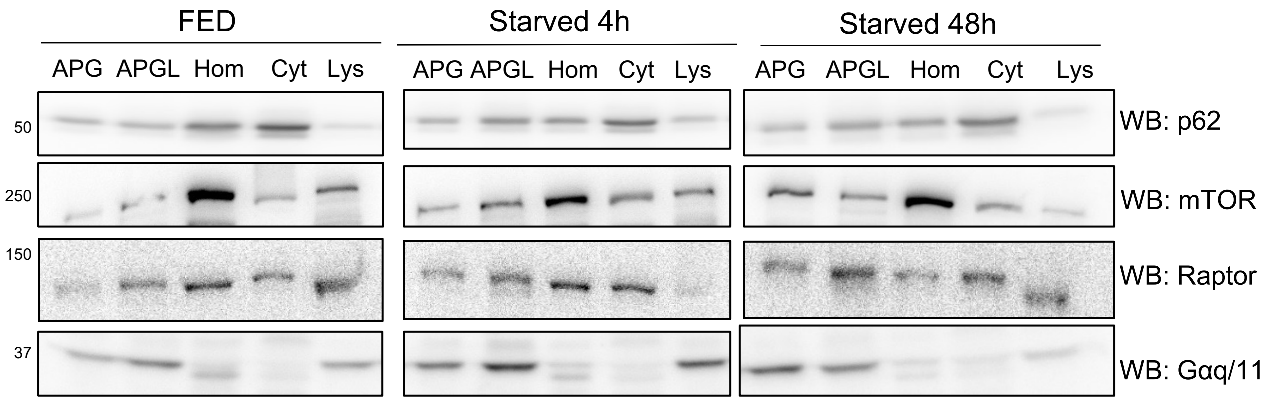

Supplementary Figure 1

Supp Fig. 2

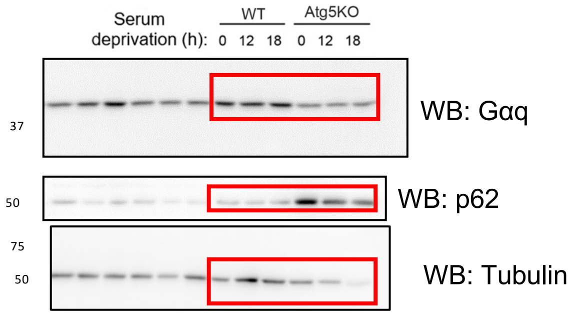

Supplementary Figure 2

Supp. Fig. 3

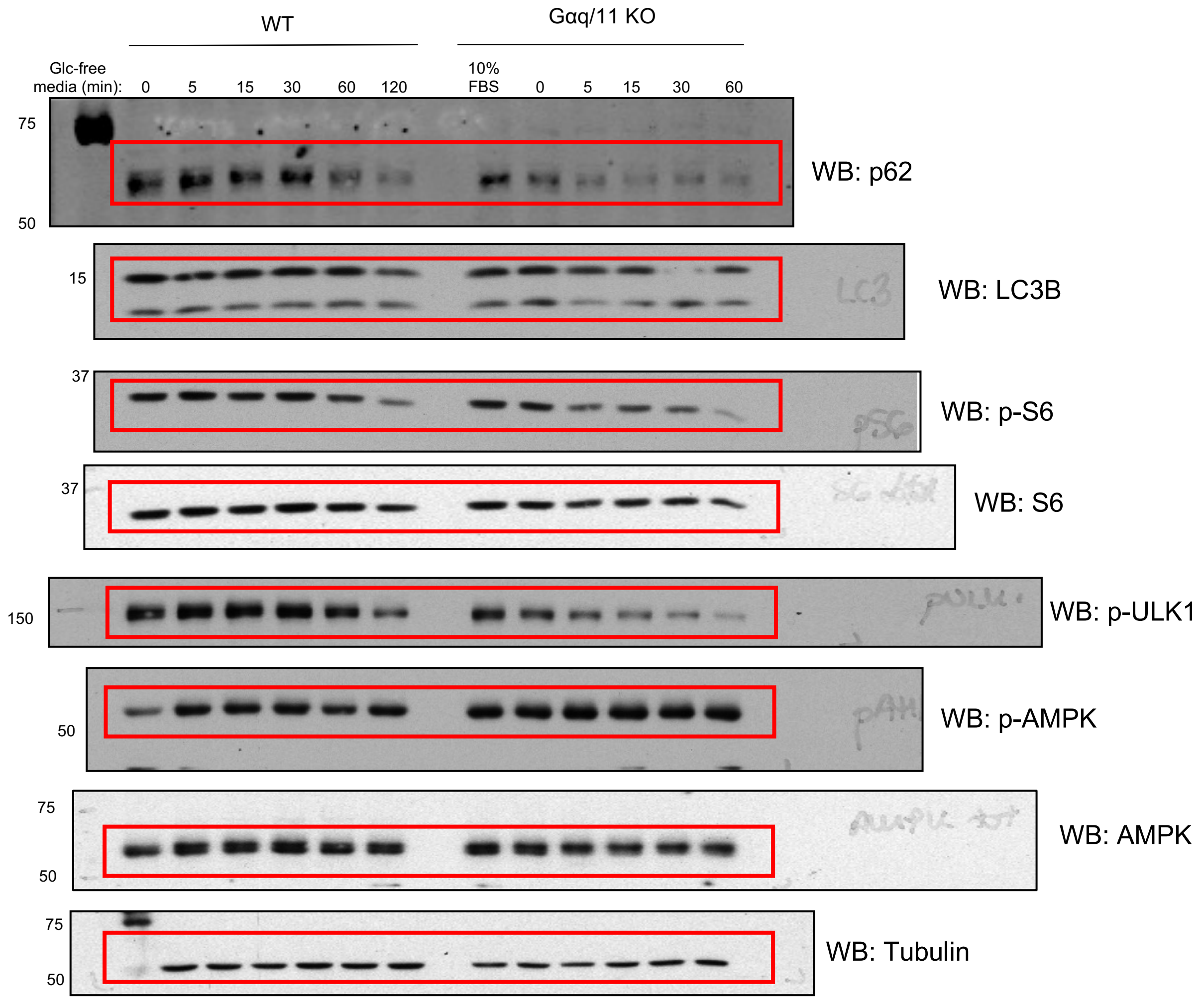

Supplementary Figure 3

Supp. Fig.6a

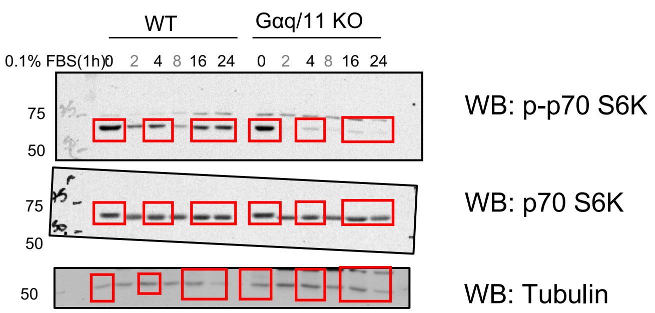

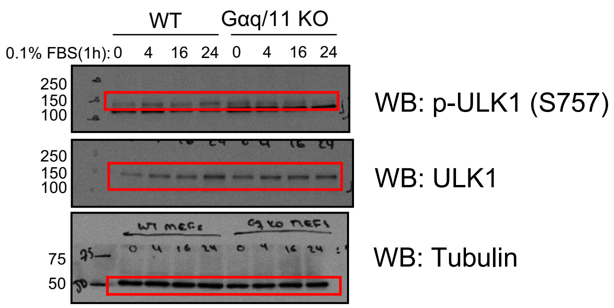

Supp. Fig.6b

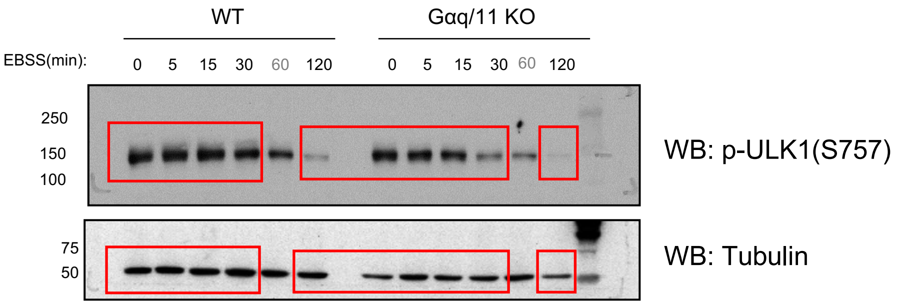

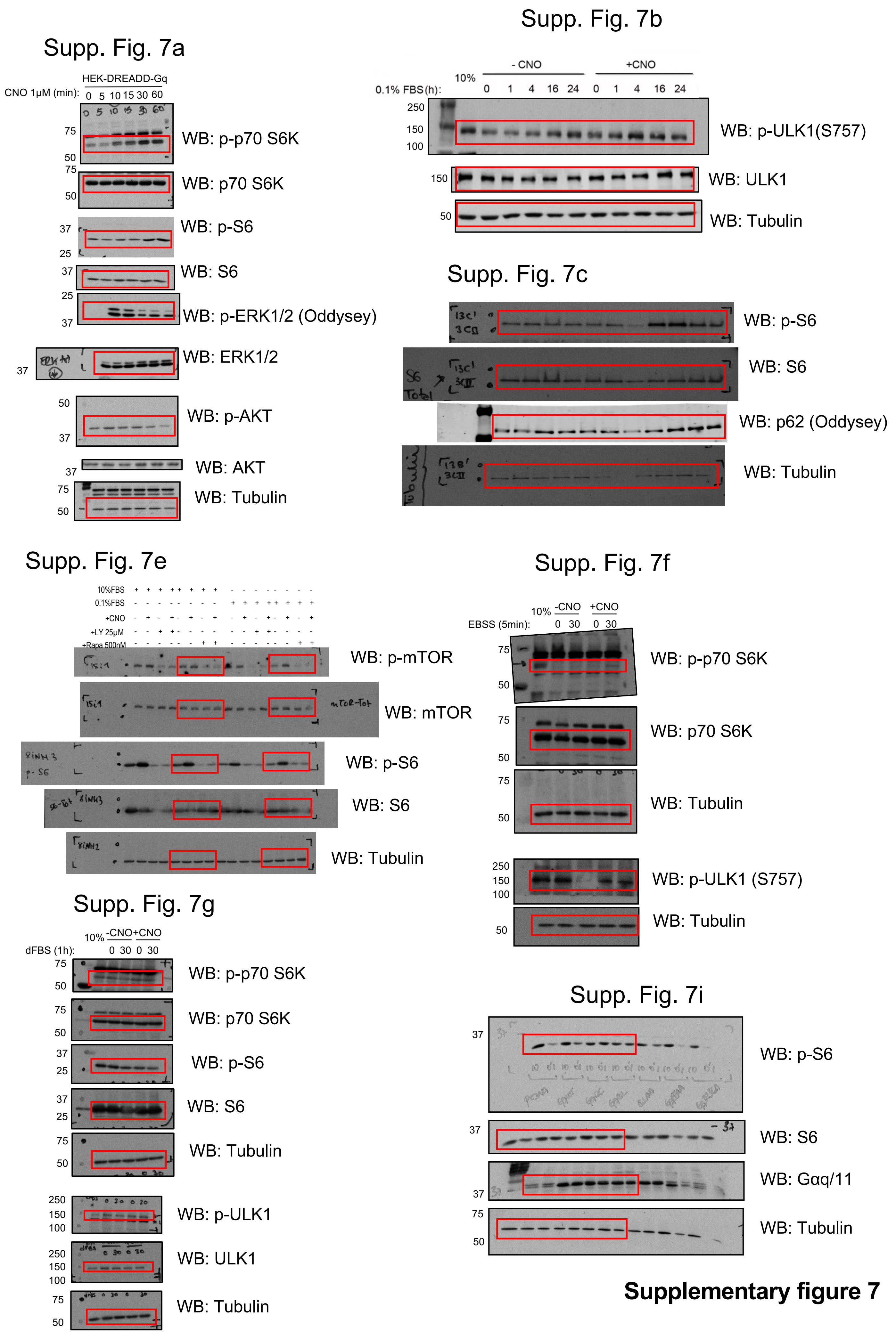

Supplementary figure 7

Supp. Fig. 8b

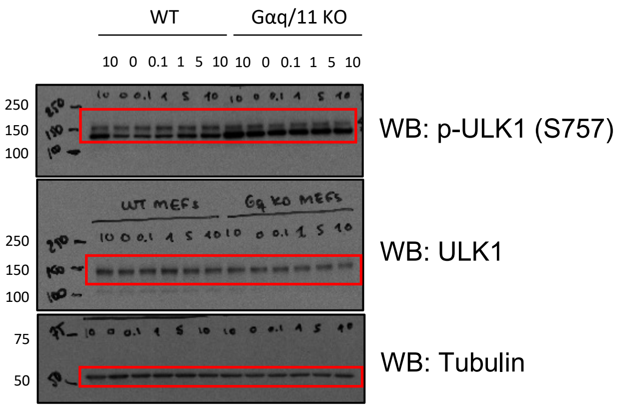

Supp. Fig. 8d

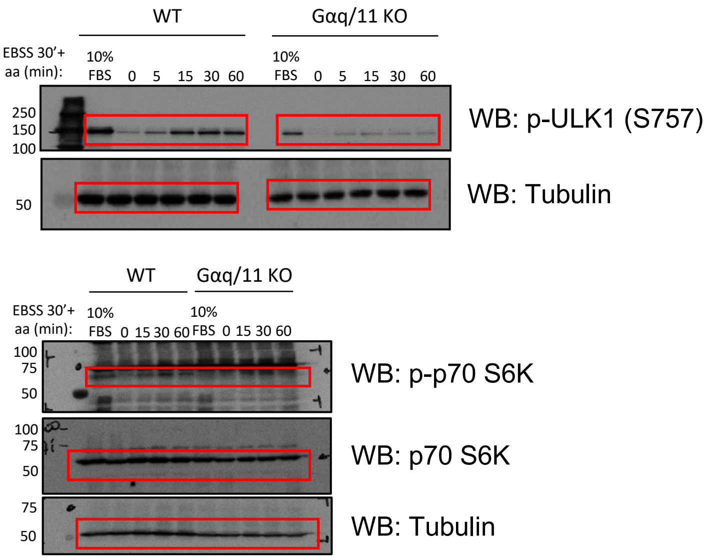

Supp. Fig. 8e

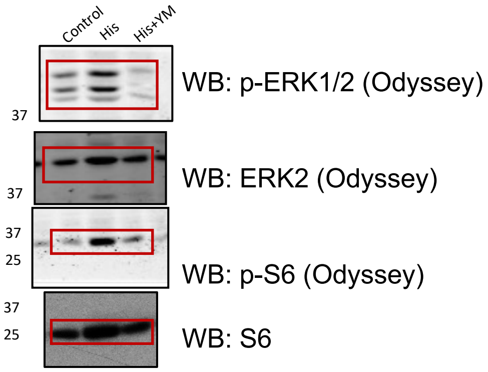

Supplementary Figure 8

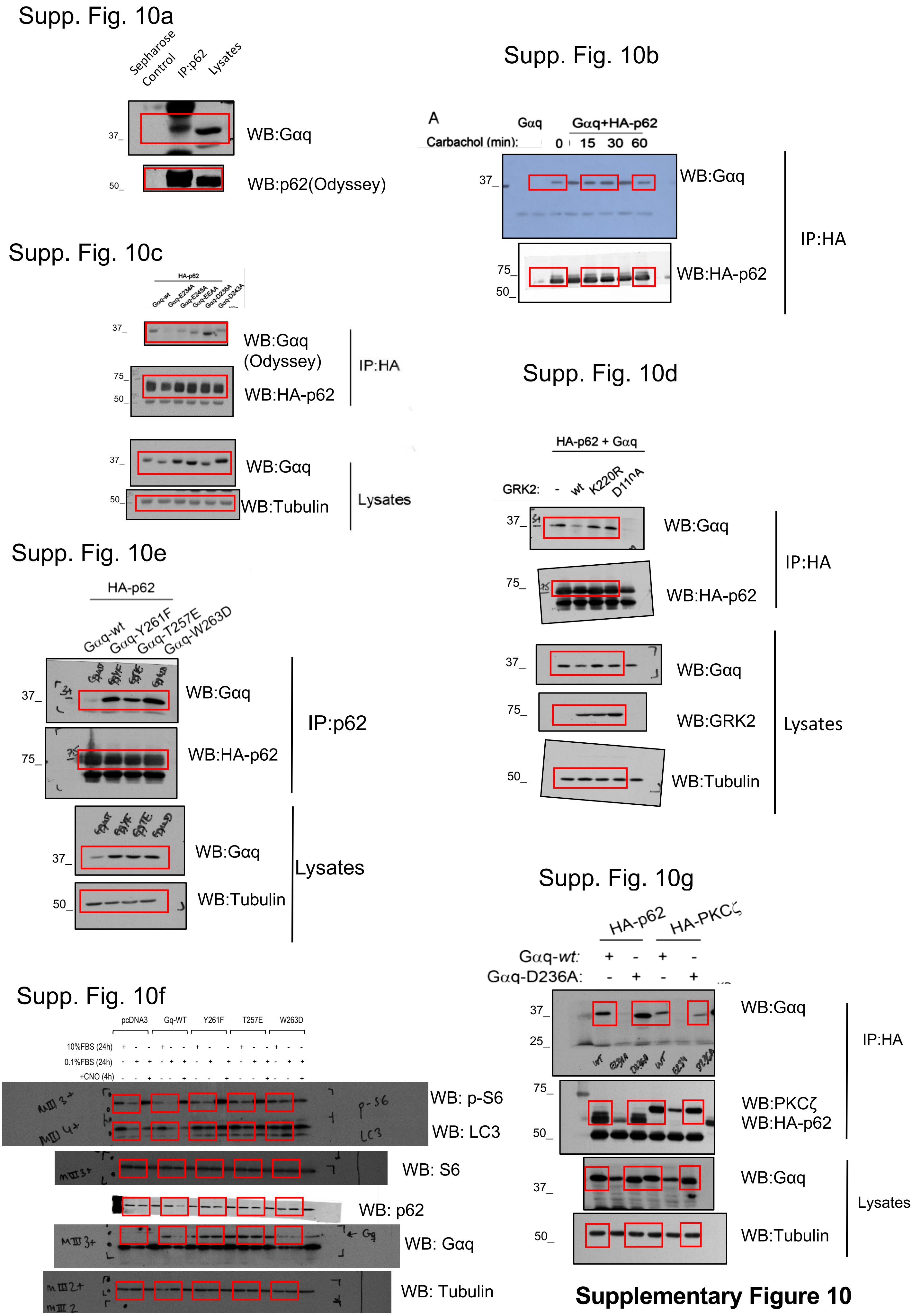

Supplementary Figure 10

Supp. Fig. 11a

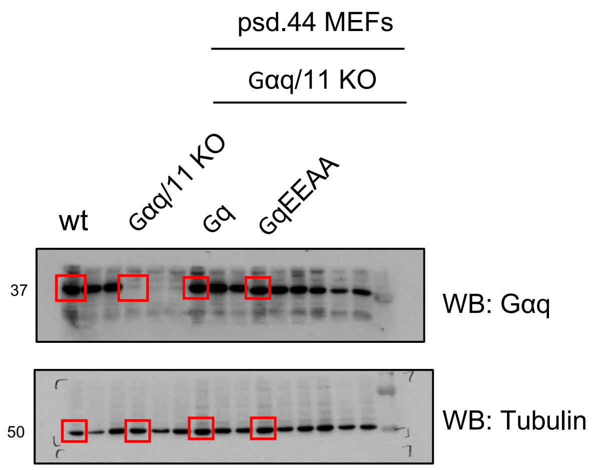

Supp. Fig. 11b

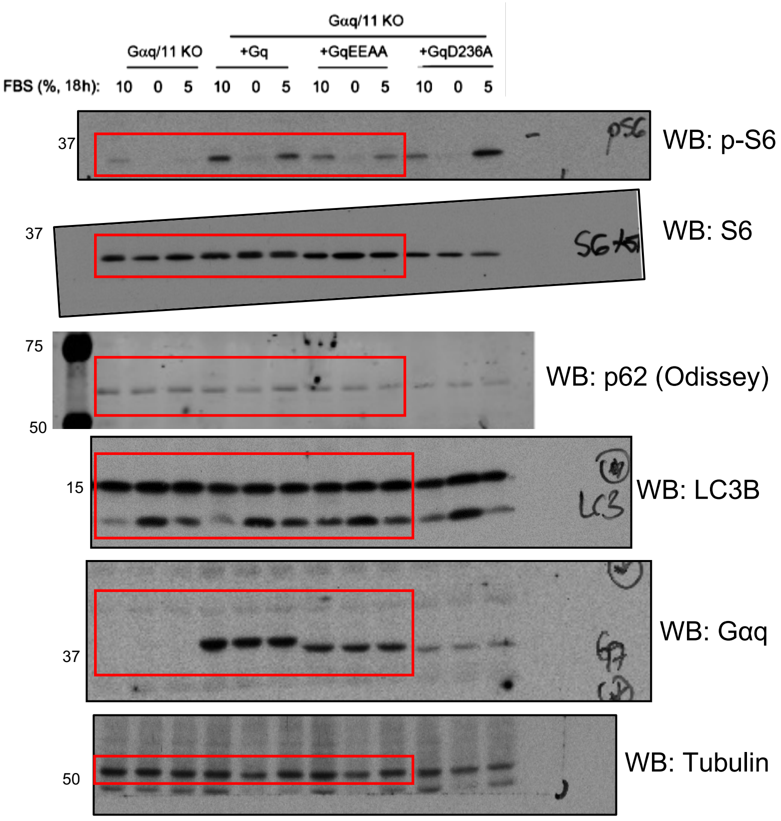

Supplementary Figure 11
